# Measures of physical functioning in adults with brain tumor associated with functional outcomes: A scoping review

**DOI:** 10.1093/nop/npaf036

**Published:** 2025-03-26

**Authors:** Tara S Davis, Emory Hsieh, Bennett A McIver, Kaitlynn Slattery, McKenzie C Kauss, Diane Cooper, Vivian A Guedes, Terri S Armstrong, Michelle L Wright

**Affiliations:** Neuro-Oncology Branch, Center for Cancer Research, National Cancer Institute, National Institutes of Health, Bethesda, Maryland, United States; Neuro-Oncology Branch, Center for Cancer Research, National Cancer Institute, National Institutes of Health, Bethesda, Maryland, United States; Neuro-Oncology Branch, Center for Cancer Research, National Cancer Institute, National Institutes of Health, Bethesda, Maryland, United States; Neuro-Oncology Branch, Center for Cancer Research, National Cancer Institute, National Institutes of Health, Bethesda, Maryland, United States; Neuro-Oncology Branch, Center for Cancer Research, National Cancer Institute, National Institutes of Health, Bethesda, Maryland, United States; National Institutes of Health Library, Office of Research Services National Institutes of Health, Bethesda, Maryland, United States; Neuro-Oncology Branch, Center for Cancer Research, National Cancer Institute, National Institutes of Health, Bethesda, Maryland, United States; Neuro-Oncology Branch, Center for Cancer Research, National Cancer Institute, National Institutes of Health, Bethesda, Maryland, United States; Neuro-Oncology Branch, Center for Cancer Research, National Cancer Institute, National Institutes of Health, Bethesda, Maryland, United States

**Keywords:** brain neoplasms, functional status, patient outcome assessment

## Abstract

Neuro-oncology researchers and clinicians rely mostly on subjective measures to evaluate physical functioning (PF) and predict survival in primary brain tumor (PBT) patients. Exploring alternative clinical outcome assessment (COA) measures may identify more objective measures that better quantify PF in PBT patients. A scoping review was conducted to identify studies related to PF measures used in PBT patients. Using the PRISMA-SCRA guideline 3 databases (PubMed, Web of Science, and Cochrane Library) were searched on January 25, 2024. Reviewers performed an independent review of titles, abstracts, and full text using covidence systematic review software and a standardized Microsoft Excel form for extracting data. 1093 publications were identified; 49 studies met eligibility criteria. Studies used a variety of PF measures evaluated at different time points, ranging from preintervention to 3 years or more postintervention. 39 PF COA measures were identified. Of the 39, 3 clinician-reported measures (ClinRO) [KPS, ECOG, and FIM] are validated for PBT. Many measures found are standardized for other neurological diseases including performance (PerfO) and patient-reported outcome (PRO) measures. Validation of additional COA types (PerfO and PRO) that are complementary to the ClinRO measures already validated for the PBT population should be established. Measures of interest should include the evaluation of walking due to its clinical relevance and indication for overall PF.

**Key Points**

- Future studies should consider establishing validation of PF COA measures that are validated in other neurological diseases for PBT patients.
- A focus should be emphasized on COA measures that evaluate walking because of its clinical importance.

In adults with primary brain tumors (PBTs) overall survival, disease progression, or response to imaging has traditionally been the primary outcomes used for both clinical care and research. In recent years a focus on patient-centered care has grown including awareness of the need to better quantify and measure the magnitude of how a patient feels, functions, or survives through the use of clinical outcomes assessments (COAs).^1–3^ Appropriate utility of COAs is so critical that the FDA recently issued a draft guide for selecting, developing, or modifying COAs.^3^

However, despite advances in treatment, patients with central nervous system cancers^4–6^ are often left with residual symptoms and neurological deficits, resulting in reduced physical functioning (PF).^7,8^ PBT patients are highly prone to a constellation of symptoms (ie physical, cognitive, and emotional) which are likely caused by the tumor and cancer-related treatments.^7^ Physical functioning impairments are often the consequence of these symptoms^8,9^ and may present similarly to an aging individual in the general population, affecting independence, and ability to perform activities of daily living, and subsequently reducing the quality of life.^10^ The field of neuro-oncology also recognizes the need to identify relevant physical functioning measures. Recently a working group consisting of members at the FDA, NCI, and the response assessment in neuro-oncology (RANO) collectively identified walking as one of the prioritized concepts for patient care and treatment evaluation in patients with gliomas through the disease trajectory and survival.^9^

PF is defined as “the ability to carry out day-to-day activities that require physical effort”^11^ and can be impacted by both the symptoms related to a patient’s underlying cancer as well as treatment-related toxicity.^11^ PF along with disease-related symptoms and toxicity from treatment are core outcomes identified by the FDA to inform the efficacy, safety and tolerability of cancer therapy. Currently in practice, neuro-oncology researchers and clinicians rely mostly on subjective clinician-reported measures (ClinROs) (eg Karnofsky performance status scale [KPS] and Eastern cooperative oncology group performance status scale [ECOG]) to evaluate PF and predict survival in PBT patients. While these measures are beneficial, limitations do exist,^12^ and complementary COA measurement approaches including objective performance measures (eg PerfOs and use of wearables) and those that capture the patient’s own report (patient-reported outcomes or PROs) are available to assure a comprehensive and patient-centered evaluation.^11^

The primary aim of this scoping review was to identify publications using PF COA measures to evaluate PF including independence in personal activities of daily living in adults with a PBT. Our intention was to explore PF as a primary outcome and understand which COA measures are commonly used in the PBT population, identify additional useful measures that better quantify PF and may allow for more accurate prognostication of patients, and lastly, identify measures that require further validation for use.

## Methods

This review followed the general guidelines published by the Preferred Reporting Items for Systematic Reviews and Meta-Analyses extension for Scoping Reviews^13^ (Supplemental Table 1).

### Study Eligibility Criteria

The PICO framework^14^ was used to define the inclusion and exclusion criteria of the studies reviewed. The inclusion criteria applied during the selection process were (a) *population*: adults (≥18 years of age) with a PBT, (b) *intervention and comparison*: identify objective or subjective measures specific to physical functioning, (c) *outcome*: the primary outcome was physical functioning capabilities, including independence in personal activities of daily living, and (d) *study type*: only quantitative studies (descriptive or analytic) were included. Studies were excluded if (a) full texts were not available, (b) written in any language other than English, (c) pediatric patients, or (d) brain metastases patients were included, and (e) if the physical functioning measures used were nested in a measure evaluating health-related quality of life or used as part of a secondary outcome in therapeutic clinical trials. Health-related quality-of-life measures will often include nested physical functioning measures in addition to other quality-of-life measures and were outside of the scope of this review.

### Literature Search Strategy

A search of PubMed, Web of Science (Clarivate), and Cochrane Library databases was performed by an expert medical research librarian on January 25, 2024, with no publication year limitation. The finalized search strategy was created with an expert librarian who helped choose mesh terms and all mesh terms used were inclusive. All search terms related to “malignant brain tumor,” “physical functioning measures,” and “physical functioning” were used in the search strategy. The full search strategy for each scientific database is listed in Supplemental Table 2.

### Study Selection

Results were downloaded into EndNote 21.3 and then imported into Covidence systematic review software (Veritas Health Innovation www.covidence.org), and duplicates were removed. Six reviewers (B.A.M., E.H., K.S., M.C.K., M.L.W., and T.S.D.) performed a dual independent review of titles and abstracts of the articles in Phase I, title, and abstract screening for eligibility. Discordant articles for study inclusion were adjudicated through an independent tie-breaking author (M.L.W. or T.S.D.). Studies that met the criteria in Phase I moved on to Phase II, full text screening for independent review.

### Data Extraction

Data was extracted by the same 6 authors (B.A.M., E.H., K.S., M.C.K., M.L.W., and T.S.D.) for the final sample of articles using a standardized data collection form using Microsoft Excel in Phase III, including (1) *study and sample characteristics* which included the country where the study was conducted, whether the study was at a single or multicenter, study objectives, design, sample size, and participant characteristics (ie tumor type(s), age, sex, race, and ethnicity); (2) *PF measures* including which PF measure(s) were used, type(s) of PF measure or COA (ie ClinRO, PRO, and PerfO), and when measures were used (*preintervention*, *acute phase postintervention*, *subacute phase postintervention*, and *survivorship phase*); (3) *key findings of the study* in the context of PF as an outcome.

### Research Synthesis

#### COA

Results were synthesized according to types of COAs (ClinRO, Observer-Reported Outcome [ObsRO^2]^, PerfO, and PRO).^2^ ClinRO measures are reported by a trained health care professional following assessment of a patient’s health where clinical judgement, interpretation of observable signs, behaviors, or other measurable concepts related to a patient’s condition is measured.^2^ ObsRO measures are based on a report of observable signs, events, behaviors related to a patient’s health condition and are typically reported by a parent, caregiver, or someone else who is with a patient who cannot report for themselves (eg infants and patients who are cognitively impaired).^2^ PerfO measures assess a patient’s performance of a standardized task according to specific instructions by a trained individual.^2^ However, unlike ClinRO measures the administer does not apply interpretation to the scoring.^2^ Lastly, PRO measures are a patient’s evaluation of their own condition without amendment or interpretation of the patient’s response by a clinician or anyone else.^2^ Symptoms, or other unobservable perceptions only known by the patient are measured using PROs and also include the patient’s own perspective on their own physical function or activities that may be observable by others.^2^

Type of COA for each measure identified in this review was confirmed using the ePROVIDE By Mapi Research Trust platform (https://eprovide.mapi-trust.org/), a platform that provides online support for COAs, to search the Patient-Reported Outcome and Quality of Life Instruments Database (PROQOLID), a resource for COA descriptions.^15^ When the COA type was not found using ePROVIDE By Mapi Research Trust a literature search was performed for measure type and description confirmation.

#### Time of COA Measurement

For clarity and synthesis of this study time of the COA measurements was collapsed into 4 different periods of time, the *preintervention phase*, *acute phase postintervention*, *subacute phase postintervention*, and *survivorship phase* to facilitate synthesis of data. The *acute phase* was defined as time starting directly following the intervention (ie treatment, surgery, and rehabilitation) until just before 6 weeks, the *subacute phase* started at 6 weeks until just before 3 years, and the *survivorship phase* was at 3 years or longer.

#### Statistically Significant Effect of COA Measurement

Another point of interest was whether the COA measures demonstrated a statistically significant effect. To determine if a statistically significant effect was present only analytical studies that had specific time point information and used hypothesis statistical testing to calculate a *P* value was evaluated for a statistically significant effect.

## Results

### Study Selection

**Figure 1.**
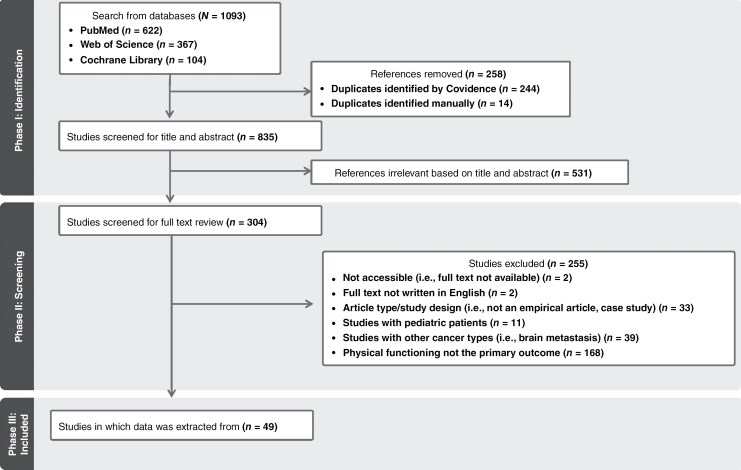
PRISMA diagram of search strategy.

Results included 1093 publications (PubMed [*n* = 622], Web of Science [*n *= 367], Cochrane Library [*n *= 104]). Covidence was used to identify and remove 244 duplicated articles, and 13 additional duplicated articles were identified and removed manually leaving 836 publications for Phase I, Title and Abstract Screening. In Phase II, 305 articles were evaluated for Full Text Screening. This process is visualized in the PRISMA flow diagram in Figure 1, and a summary of the articles extracted is presented in Supplemental Table 3.

### General Study Characteristics

Most studies were published within the past 10 years between 2014 and 2024 (*n* = 31, 63%).^16–46^ Studies were mostly conducted in North America (United States, *n* = 11 (22%),^17,34,37,39,47–53^ Canada, *n* = 4 (8%)^21,36,41,44^), and the majority were performed at single centers (*n* = 45, 92%).^16–21,23–43,45–52,54–63^

### Study Participant Characteristics

Collectively all 49 studies consisted of 8153 individuals with the study sample sizes ranging from 10 to 1660, a median sample size of 77 and an average sample size of only 167, with over 50% of studies reporting a sample size of less than 100 (*n* = 29, 60%).^16,17,21–24,27–30,32,33,36,38,41–45,49,51,52,54,55,57–59,62,64^ On average, studies had more male (57%) than female participants and ages ranged from 18 to 89 years of age with a mean and median age of 51 and 62, respectively. Very few studies reported race or ethnicity (*n* = 5, 10%),^34,39,48,49,53^ with White and non-Hispanic most reported. A third of the studies (*n* = 15, 31%) were conducted in patients with 1 tumor type, most commonly glioblastoma (*n* = 11, 22%),^18,20,23,28,29,31,32,37,38,47,57^ and 4 (8%) studies only included meningioma tumors.^17,26,33,64^ Over half (*n* = 28, 57%) of the studies focused solely on glioma tumor types.^16,18–24,27–32,35–38,44–47,50,51,55–57^

### Study Design

**Figure 2.**
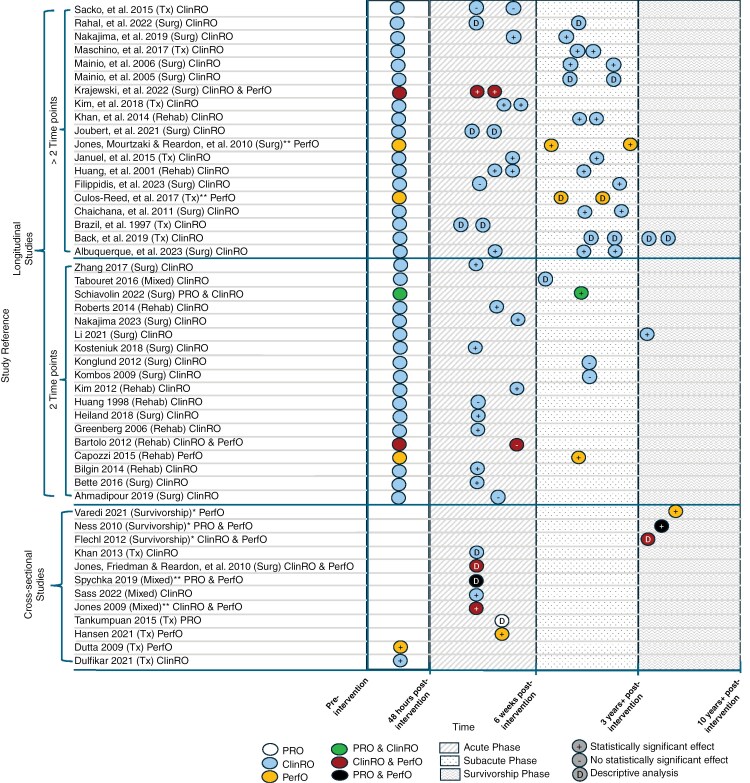
Jitter plot of study timepoints and if a statistical effect was present in each study (*N* = 49). *Indicates adult survivors of childhood brain cancer/long-term survivors of brain cancer study. **Indicates feasibility study that tested the feasibility of COA measure(s). Treatment intervention (Tx), clinical-reported outcome (ClinRO), surgical intervention (Surg), performance outcome (PerfO), rehabilitation intervention (Rehab), mixed interventions (Mixed), and patient-reported outcome (PRO).

Only 4 (10%) studies aimed to assess measurement feasibility.^36,41,50,52^ Nearly all studies were observational apart from 1 study that was a clinical controlled trial.^65^ A total of 29 (59%) studies were prospective,^16,18,19,21,22,24,25,27,34,36,39,40,43,48–59,61–64^ of those studies, 10 (20%) used a cross-sectional design to measure PF at 1-time point,^22,34,39,43,50,51,53,57,61,63^ 19 (39%) used a longitudinal approach^16,18,19,21,24,25,27,36,40,48,49,52,54–56,58,59,62,64^ (Figure 2) and 5 (10%) applied a case-control design.^25,48,53–55^ Nineteen (39%) studies were retrospective,^17,20,23,26,28–33,35,37,38,41,42,44,45,47,60^ of those studies, 2 (4%) used a cross-sectional model,^30,41^ 17 (35%) used a longitudinal method^17,20,23,26,28,29,31–33,35,37,38,42,44,45,47,60^ and 3 (6%) included a case-control design.^44,45,60^ Of the 8 total (16%) studies that used a case–control design^25,44,45,48,53–55,60^ 1 retrospective propensity-matched study compared outcomes (including postoperative functional status) in low-grade glioma patients who underwent preoperative functional magnetic resonance imaging (fMRI) compared to a control group of low-grade glioma patients who did not undergo a preoperative fMRI.^55^ Another study compared patient outcomes (including functional outcomes) in 2 different groups of patients with a glioma, not adjacent to the motor cortical areas without the use of intraoperative monitoring compared to patients with a glioma adjacent to the central region operated under intraoperative monitor.^55^ However, most case–control studies used control groups consisting of ischemic or hemorrhagic stroke patients (*n* = 3),^48,54,60^ 2 studies used general population-based control groups,^25,53^ and 1 study used traumatic brain injury patients as controls.^45^

### PF COAs Identified in the Sample

**Figure 3.**
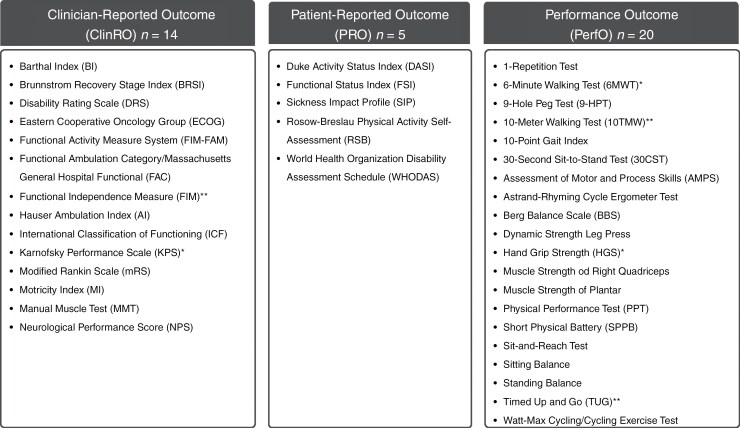
Physical functioning measures identified and listed by type of COA measure (*N* = 39). *Karnofsky Performance Scale (KPS) was the most used clinician-reported measure (*n* = 31); **Functional Independence Measure (FIM) were the second most used clinician-reported measure (*n* = 6); *Hand Grip Strength was the most used performance outcome measure (*n* = 4); and the **6-Min Walking Test and the **10-M Walking Test (*n* = 3) were the second most used performance outcome measure. Each patient-reported outcome measure identified in this sample was only used once.

Three types of PF COA measures were identified including 20 PerfOs, 14 ClinROs, and 5 PROs (Figure 3). This review did not identify any ObsRO measures. The most common PF domain identified across all COAs evaluated was activities of daily living (Supplemental Table 4). Activities of daily living were also the most common domain measured in both ClinRO (*n* = 10) and PRO (*n* = 4) measures (Supplemental Tables 5 and 6), and muscle strength was the most common PerfO domain (*n* = 7) measured (Supplemental Table 7). Three COA measures found in this review have been standardized for evaluating PF in the PBT population (ie KPS, ECOG, and FIM). The measures KPS, ECOG, and FIM have been standardized meaning that these measures have been validated and are widely used in the PBT population (Supplemental Table 5).

#### ClinRO COA Measures

Most articles used at least 1 ClinRO (*n* = 41, 84%)^16–20,23–29,31–33,35,37–40,42,44–52,54–64^ (Figure 3) and over half of the studies measured PF using KPS (*n* = 31, 63%).^16–18,20,23–29,31–33,35,38–40,42,44,47,49–51,55,56,58,59,62–64^ The functional independence measure (FIM) (*n* = 6, 12%)^37,46,48,49,54,60^ and Barthel Index (BI) (*n* = 6, 12%)^25,27,45,56–58^ were the next most used ClinRO measures (Figure 3).

**Figure 4.**
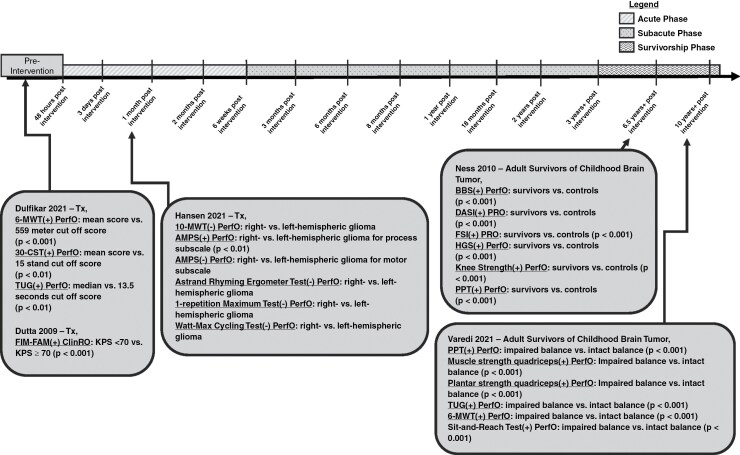
Timeline of cross-sectional analytical studies with detailed time point information. Studies were only included in the timeline if specific time point information was given and if it was an analytical study (not descriptive). (+) Indicates a statistical effect; (–) indicates no statistical effect. Treatment (Tx); 6-Min Walking Test (6-MWT); 30-Sit-To-Stand Test (30-CST); Timed-Up-and-Go Test (TUG); Functional Measure System (FIM–FAM); 10-Min Walking Test (10-MWT); Assessment of Motor and Process Skills (AMPS); Berg Balance Scale (BBS); Duke Activity Status Index (DASI); Functional Status Index (FSI); Hand Grip Strength (HGS); Physical Performance Test (PPT).

**Figure 5.**
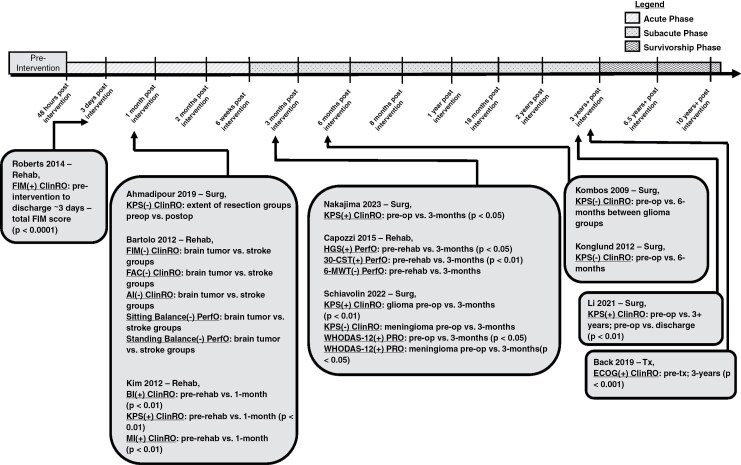
Timeline of longitudinal analytical studies with 2-time points (preintervention and an additional time point). Studies were only included in the timeline if specific time point information was given and if it was an analytical study (not descriptive). Each study evaluated physical functioning preintervention and postintervention (Rehab; Surg; Tx). (+) indicates a statistical effect; (–) indicates no statistical effect. Rehabilitation (Rehab); prerehabilitation (prerehab); surgery (Surg); preoperative (preop); functional independence measure (FIM); Karnofsky performance status scale (KPS); functional ambulation category (FAC); Barthal index (BI); motricity index (MI); hand grip strength (HGS); 30-sit-to-stand test (30-CST); 6-min walking test (6-MWT); World Health Organization Disability Assessment Schedule (WHODAS-12); Eastern Cooperative Oncology Group (ECOG).

**Figure 6.**
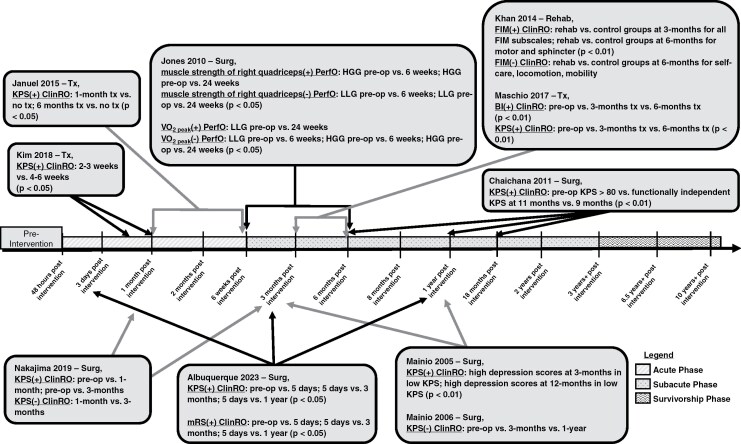
Timeline of longitudinal analytical studies with more than 2-time points (preintervention and 2 or more additional time points). Studies were only included in the timeline if specific time point information was given and if it was an analytical study (not descriptive). Each study evaluated physical functioning preintervention and at least 2 additional postintervention (Rehab, Rehabilitation; Surg, Surgery; Tx, Treatment) time points. (+) indicates a statistical effect; (–) indicates no statistical effect. High-grade glioma (HGG); low-grade glioma (LGG); Karnofsky performance status scale (KPS); Astrand–Rhyming cycle Ergometer test/maximum or peak oxygen volume (VO_2max/peak_); functional independence measure (FIM); Barthal index (BI); modified rankin scale (mRS). Each study evaluated physical functioning preintervention and multiple time points postintervention (Rehab, rehabilitation; Surg, surgery; Tx, treatment).

A total of 20 (41%) analytical studies used at least 1 ClinRO measure and provided specific time point information (Figures 4–6).^16,18,19,24,26–29,32,37,40,46,47,54,55,58,59,62–64^ Of the 20 analytical studies, 8 longitudinal studies included at least 1 ClinRO evaluation at the *acute phase* (Figures 5 and 6).^16,18,24,29,32,37,54,58^ Most analytical studies utilizing at least 1 ClinRO measure (n = 12) were longitudinal and included at least 1-time point during the *subacute phase* (Figures 5 and 6),^16,27–29,32,40,46,47,55,59,62,64^ 2 studies included at least 1 time point in the *survivorship phase* (Figure 5),^19,26^ and 1 cross-sectional study used ClinRO measures only in the *pretreatment phase* (Figure 4).^63^

The most frequent ClinRO measure among the analytical studies with specific time point information was KPS (*n* = 15), and was used at various time points ranging from preintervention to 3 or more years (Figures 5 and 6).^16,18,24,26–29,32,40,47,55,58,59,62,64^ Of the fifteen studies that used KPS, 10 (67%) were found to have a statistically significant effect in at least 1-time point across the *acute*, *subacute*, and *survivorship phases* (Figures 5 and 6).^16,24,26–29,32,47,58,59^

Three analytical studies used the FIM,^37,46,54^ and of those studies, 2 had a statistically significant effect at least at 1 time point(Figures 5 and 6).^37,46^ FIM was measured at different time points ranging from the *acute* to *subacute phases*. Roberts et al. had a statistically significant effect when comparing PF using the FIM before rehabilitation and discharge from rehabilitation in the *acute phase*. Khan et al. also had a statistically significant effect comparing a rehabilitation group with a control group not receiving rehabilitation in the *subacute phases* for total FIM and FIM motor and sphincter subscales (Figure 6). Whereas Bartolo et al. did not have a statistically significant effect, however, this study tested differences in PF using the FIM to compare brain tumor patients and a control group of stroke patients before-rehabilitation and postrehabilitation in the *acute phase* (Figure 5).

Two analytical studies used the Barthal index (BI) to evaluate PF.^27,58^ Kim et al. had a statistically significant effect using the BI to compare before rehabilitation and postrehabilitation in the *acute phase*^58^ (Figure 5). Maschio et al. also had a statistically significant effect comparing BI before surgery and postsurgery in *subacute phases*^27^ (Figure 6).

One analytical cross-sectional study used the Functional Activity Measure System (FIM–FAM), an expanded version of the FIM, to measure PF during the *preintervention phase*.^63^ Two different analytical studies used the Functional Ambulation Category (FAC)^54^ and Motricity Index (MI)^58^ during the *acute phase* (Figure 5). The FAC measure did not have a statistically significant effect,^54^ however the MI did have a statistically significant effect (Figure 5).^58^ One study used the Eastern Cooperative Oncology Group Performance Status Scale (ECOG) to compare physical function at *pretreatment* and the *survivorship phases* and had a statistically significant effect (Figure 5).^19^

#### PerfO COA Measures

Of all 49 articles, 13 (27%)^21,22,25,30,34,36,41,50–54,57^ used at least 1 PerfO measure (Figure 2). The most commonly used PerfO measures included hand grip strength [HGS] (*n* = 4, 8%),^21,36,41,53^ 6-min walking test [6-MWT] (*n* = 4, 6%),^21,22,34,50^10-m walking test [10-MWT] (*n* = 2, 4%)^30,57^ and timed-up-and-go test [TUG] (*n* = 2, 4%) (Figure 3).^22,34^

A total of 7 analytical studies that used at least 1 PerfO measure and provided specific time point information were identified (Figures 4–6).^21,22,30,34,52–54^ Two studies used PerfO measures in at least 1 evaluation during the *acute phase* (Figures 4 and 5).^30,54^ In Hansen et al. the only statistically significant effect found was in the assessment of motor and process skills (AMPS) measure but only for the processing subscale comparing glioma location (right vs. left-sided tumor) during the *acute phase* time point.^30^ Whereas the 10-MWT, AMPS motor subscale, Astrand Rhyming Ergometer Test, 1-repetition maximum test (1-RMT), and Watt–Max cycling test did not have a statistically significant effect during the *acute phase* (Figure 4).^30^ In Bartolo et al. the only 2 PerfO measures used were sitting balance and standing balance in the *acute phase*, and neither had a statistically significant effect when comparing balance in brain tumor patients and a control group consisting of stroke patients (Figure 5).^54^

Two analytical studies used PerfO measures during the *subacute phase*.^21,52^ In Capozzi et al. and Jones et al. PerfO measures were the only type of COAs used to assess PF during the *subacute phase* (Figures 5 and 6).^21,52^ Capozzi et al. used HGS, 30-Sec Sit-to-Stand Test (30-CST) and 6-MWT before rehabilitation and *subacutely* postrehabilitation. There was a statistically significant effect for HGS and 30-CST, however there was no statistically significant effect for the 6-MWT (Figure 5).^21^ Whereas Jones et al. assessed quadriceps muscle strength and peak oxygen volume before surgery at 2 additional time points during the *subacute phase*. There were statistically significant effects when comparing quadriceps strength in high-grade gliomas presurgical, and 2 additional time points in the *subacute phase,* however, there was no effect for quadricep strength in low-grade glioma patients (Figure 6).^52^

Two studies assessing PF in adult survivors of childhood brain cancer used PerfO measures during the *survivorship phases* (Figure 4).^34,53^ Ness et al. used BBS (Berg Balance Scale), HGS, Knee strength, and PPT. All 4 measures had a statistically significant effect when comparing brain tumor survivors and a group of controls from the general population^53^ (Figure 4). Varedi et al. stratified balance to compare impaired balance versus intact balance to study PF in adult survivors of childhood brain cancer and used 6 PF measures (ie 6-MWT, muscle strength quadriceps, plantar strength quadriceps, PPT, Sit-and-Reach Test, and TUG) all of which had a statistically significant effect^34^ (Figure 4). Lastly, a cross-sectional study by Dulfikar and colleagues evaluated 3 PerfO measures (ie 6-MWT, 30-CST, and TUG) before treatment, and all 3 measures had a statistically significant effect (Figure 4).^22^

#### PRO COA Measures

PRO measures were used the least. Each PRO identified was only used once and included the Duke activity status index (DASI),^53^ functional status index (FSI),^53^ sickness impact profile (SIP),^43^ Rosow–Breslau physical activity self-assessment,^41^ and the World Health Organization disability assessment schedule (WHODAS)^40^ (Figure 3).

Two analytical studies with time point information used PRO measures.^40,53^ First, Schiavolin et al. used the WHODAS in a longitudinal design to evaluate PF before surgery and in the *subacute phase*, which did have a statistically significant effect^40^ (Figure 5). The second study was a cross-sectional study by Ness et al. using both the DASI and FSI PRO measures to compare adult survivors of childhood brain cancer with a control group made up of the general population in the *survivorship phase*.^53^ Both PRO measures had a statistically significant effect (Figure 4).^40,53^

## Discussion

In this scoping review, the literature was reviewed for studies using COA measures to evaluate PF in PBT patients. Only 3 COA measures identified, all of which are ClinRO measures, in this review have been standardized for evaluating PF in the PBT population (ie KPS, ECOG, and FIM). Several COA PF measures were identified that have not been standardized for the PBT population but were used to evaluate PBT patients during various stages of their cancer. On the other hand, many of these COA measures have been standardized in other neurological diseases (ie stroke [BI, FIM, mRS, SIP, 9-HPT, 10-TMW, and BBS], traumatic brain injury [DRS and FIM], spinal cord injury [FIM and 10-TMW]). Other interesting findings included the 3 case studies evaluating PF in PBT patients compared with stroke patients,^48,54,60^ and an additional case study comparing PBT patients with traumatic brain injury patients.^45^

Pathologically, patients with brain tumors, strokes, and traumatic brain injuries are very different, however, often these patients may present clinically similar. These diseases often cause some type of brain injury (nontraumatic or traumatic). Depending on the severity of the disease, intervention, and tumor or lesion type these brain injuries may contribute to similar symptoms, as well as cognitive and PF impairments (eg weakness, difficulty walking, and balance issues) that may require similar rehabilitation needs.^54^ Therefore, other measures already validated for patients after stroke or traumatic brain injury may also be useful for measuring other PF domains in the PBT population.

### Standardized PF COA Measures Used in Brain Tumor Patients

The ClinRO measures KPS and FIM were identified as the most important overall. These measures are widely known to be used to assess PF in oncology patients, including the brain tumor population.^66^ The ClinRO measure ECOG was only identified once in this review^31^ and is validated broadly for use in oncology patients.^67^

#### Karnofsky Performance Scale (KPS)

The most prominent COA measure in this study was the KPS (*n* = 31). The KPS is a ClinRO measure that has been widely used to measure PF in oncology since its development in 1948,^68^ both clinically and in clinical trials, as a prognostic tool to determine appropriateness for surgical intervention, drug therapy, and other nonpharmacological interventions,^66^ including physical therapies. The KPS is especially beneficial for the neuro-oncology population due to its quick and easy method for clinicians to complete with patients, its predictive power for survival and quality of life, as well as its ability to compare the effectiveness of different interventions.^69^ The KPS scale ranges from 0 to 100%, where higher scores indicate better functional status and measures a patient’s ability to perform daily tasks and functional status (Supplemental Table 5).^68^ However, the KPS comes with some limitations including possible influence from acute events, it may be a poor predictor of prognosis when a score is ≥90%, and also it is problematic for identifying small changes in PF.^68^ More broadly the KPS is subjectively based on a clinician’s observation of the patient, which may result in poor inter-rater reliability.^50,70^

#### Functional Independence Measure (FIM) and Functional Activity Measure System (FIM–FAM)

The FIM was the second most used ClinRO measure in this review (*n* = 6), however unlike the KPS and ECOG, it was developed to evaluate PF in patients undergoing physical rehabilitation.^71^ The FIM is a broadly acceptable measure used for monitoring activities of daily living with an emphasis on PF in patients with neurological conditions undergoing rehabilitation including neuro-oncology patients,^48,63^ patients following a stroke,^72^ and patients following a traumatic brain injury.^72,73^ Generally, the FIM is used within 72 h of admission and within 72 h prior to discharge from rehabilitation. Higher scores indicate a patient has more independence in performing daily tasks.^74^ Two subscales make up the FIM: the motor FIM (ie self-care, mobility, and sphincter control) and the cognitive FIM (ie communication, social interaction, and problem-solving) (Supplemental Table 5). The motor domain has been shown to be highly correlated with the BI, another ClinRO measure typically used in stroke patients.^75^ Inter-rater reliability and validity in the general inpatient rehabilitation population have shown to be good.^76^ However, 1 limitation is that scores may be affected by a patient’s environment since the measure was designed to be used during physical rehabilitation.^74^

Also important, the FIM emphasizes domains pertaining to PF, an ancillary measure, the Functional Assessment Measure (FAM) was developed to address other domains that affect physical function (eg cognition, behavior, communication, and community functioning).^63^ The FIM and Functional Assessment Measure (FAM) are together known as the Functional Activity Measure System (FIM–FAM) and have very good inter-rater reliability and validity in patients undergoing rehabilitation.^77^ Only 1 study was identified in this review that used the FIM–FAM (Supplemental Table 3a).^63^

#### Eastern Cooperative Oncology Group Performance Status Scale (ECOG)

Another ClinRO measure identified and validated in the oncology population was the ECOG, also referred to as the World Health Organization Performance Status.^78^ Adapted from the KPS, the ECOG is also useful for determining a patient’s prognosis and eligibility for interventions; also the ECOG has similar limitations to the KPS.^67^ However, different from the KPS the ECOG uses a shorter scale (0–5) which may be more efficient to complete compared to the KPS.^67^ The scoring of the ECOG scale is opposite to the KPS where lower scores indicate better PF (Supplemental Table 5).^67^ The ECOG is often used interchangeably with the KPS due to the strong correlation between the 2 measures for patients with cancer.^79^

### Additional Important Standardized COA Measure Used in PBT Patients

There are additional standardized measures for use in PBT that were not identified in this review or did not meet the study criteria due to multidimensionality including the European Organisation for Research Treatment of Cancer Core Quality of Life Questionnaire (EORTC QLQ-C30), European Organisation for Research Treatment of Cancer Core Instrumental Activities of Daily Living Questionnaire (EORTC IADL-BN32), functional assessment of cancer therapy (FACT) scale, MD Anderson Symptom Inventory Brain Tumor Module (MDASI-BT), and neurologic assessment in neuro-oncology (NANO). These COA measures are designed to measure quality of life, symptom burden, or neurologic functioning but include PF as a component of the tool and are briefly presented below. The use of these tools to measure PF may be limited due to issues of construct clarity, but use may further inform the clinical implications of the measure.

#### EORTC Core Quality of Life Questionnaire (EORTC QLQ-C30) and EORTC Instrumental Activities of Daily Living Questionnaire (EORTC IADL-BN32)

The EORTC QLQ-C30 is a 30-item COA PRO measure used to evaluate health-related quality of life in cancer patients, including PBT patients and has been validated in an international phase IV setting.^80^ The measure includes 15 scales that consist of 5 functional scales (ie physical, role, emotional, social, and cognitive) and 8 symptom scales (ie pain, fatigue, appetite loss, constipation, dyspnea, diarrhea, nausea, and vomiting). Scores range from 0 to 100 where higher scores on the global quality of life scales indicate better health, and higher scores on the symptom scales indicate more symptom burden.^81^

The EORTC IADL-BN32, a recently developed PRO COA measure for brain tumor patients, assesses basic activities of living and instrumental activities of daily living that are related to independent functioning in society (eg work and household activities) and more often requires a higher-order cognitive functioning to complete. The scores range from 0 to 100, where high scores indicate the patient has more difficulty with instrumental activities.^82^

#### *Functional Assessment of Cancer Therapy-General (FACT-G) and Functional Assessment of Cancer Therapy*-*Brain (FACT-Br) Scales.*

The FACT-G is a PRO measure with 27 items divided into 4 domains including physical well-being, social/family well-being, emotional well-being, and functional well-being in the past 7 days measured on a scale from 0 to 108 where higher scores indicate better well-being.^83^ The FACT-G is validated for any tumor type, however another measure, the FACT-Br was developed specifically for the brain tumor population and consists of the same domains from the FACT-G measure as well as a brain subscale. The brain subscale evaluates cognitive as well as neurological characteristics, and scores range from 0 to 200 where higher scores indicate better well-being.^84^

#### MD Anderson Symptom Inventory Brain Tumor Module (MDASI-BT)

Another important COA measure that has been validated in PBT patients^85^ used in our group and other groups^86^ is the MD Anderson Symptom Inventory Brain Tumor Module (MDASI-BT). The MDASI-BT is a PRO measure that assesses the severity of symptoms and consists of 22 symptom items that evaluate for affective, cognitive, neurologic, treatment-related, general disease, and gastrointestinal symptoms as well as 2 symptom interference subscales (activity- and mood-related interference) measuring symptom interference with daily activities of living.^85^ The MDASI-BT uses a Likert scale for each item ranging from 0 to 10 where higher scores indicate worse symptoms or more interference and assesses symptoms and interference within the last 24 h.^85^ Domains pertaining to PF that the MDASI evaluates include work-, walking-, and general activity-related interference.

#### Neurologic Assessment in Neuro-Oncology (NANO)

Another measure used in neuro-oncology patients is the neurologic assessment of neuro-oncology (NANO), which was created as a measure for evaluating neurological function in neuro-oncology patients. The NANO assesses 9 different domains (ie gait, strength, ataxia, sensibility, visual field, facial paralysis, language, level of consciousness, and behavior) where higher scores indicate worse neurological function.^87^

### PF COA Measures Commonly Used in Other Neurological Diseases

Of all 39 COA measures identified in this review (Figure 3) only 3 ClinRO measures (KPS, ECOG, and FIM) have been standardized for brain tumor patients (Supplemental Table 5). Eight COA measures (BI, FIM, mRS, SIP, 6-MWT, 9-HPT, 10-TMW, and BBS) (Supplemental Tables 5–7) were found in the literature to be commonly used, validated, or reliable in patients poststroke.^72,88–93^ Additionally, there were 2 COA measures (DRS and FIM) found in the literature to be commonly used, validated and reliable in traumatic brain injury patients (Supplemental Table 5).^74,94^

#### COA Measures Identified That Are Commonly Used in Patients After a Stroke

A total of 7 COA measures identified in this review were found to be commonly used, validated and reliable for use in stroke patients, 4 were PerfO measures (ie 6-MWT, 9-HPT, 10TMW, and BBS) (Supplemental Table 7), 3 were ClinRO measures (BI, FIM, and amRS) (Supplemental Table 5), and 1 was a PRO measure (SIP) (Supplemental Table 6).

The 6-MWT and 10-TMW were both commonly identified in this review. Although both measures are used to quantify a patient’s ability to walk, the 6-MWT focuses on aerobic endurance^95^ whereas the 10-TMW results are based on a patient’s walking speed.^96^ Walking assessments, like the 6-MWT and 10-TMW, should be strongly considered for validation in the brain tumor population. It is known that brain tumor patients who are safely able to perform low-intensity exercise like walking reduce risks for other chronic diseases^97^ and decrease treatment-related symptoms.^98^ Some evidence has shown that low-intensity exercise can reduce cancer recurrence and mortality^99^ while subsequently improving quality of life.^98^ Additionally, having the ability to walk safely in one’s own environment and community is important for maintaining activities of daily living independence. Often walking and other mobility limitations (eg balance) seen clinically may be the first indication of decreased physical functioning or disability, specifically when assessing the walking speed domain.^100,101^ Furthermore, pertaining to neuro-oncology, a working group including members from the FDA, NCI, and the RANO identified that walking is a priority for patient care but is also prioritized as a treatment evaluation in glioma patients.^9^ However, most importantly standardized subjective assessments like timed walking tests are critically needed in the field of neuro-oncology. Therefore, it would be important to focus on validating PerfO measures for PBT patients in the future. This review identified 4 PerfO measures that evaluated walking in PBT patients and included 6-MWT (a timed walking assessment), 10-TMW (tests walking speed in meters per second), 10-PGI (tests gait based on 10 different tasks grading gait), and the SSPB (measures lower extremity physical performance based on 3 timed tasks including walking speed). A summary of each of these PerfO walking or ambulation measures as described in Supplemental Table 7*PerfO COA Measures Identified.*

#### COA Measures Identified That Are Commonly Used in Patients After a Traumatic Brain Injury

Two ClinRO COA measures (ie DRS^94^ and FIM^74^) were identified in this review that were also found in the literature to be commonly used, validated, and reliable for use in patients following a traumatic brain injury (Supplemental Table 5). The DRS measures cognition and activities of daily living in patients with scores ranging from 0 to 30 where higher scores indicate more disability; however, a score of 30 indicates death. The DRS is also an alternative measure to the Glasgow Outcome Scale or the Extended Glasgow Outcome Scale, common scales used clinically to measure consciousness and functional outcomes respectively in patients after a traumatic brain injury.^94^ As described above the FIM is broadly validated for patients undergoing rehabilitation, including after a traumatic brain injury.^74^

### Limitations of the Study

This review does possess some limitations. First, this study limited its inclusion to adult PBT patients only. In the literature, pediatric studies utilized additional measures not mentioned in this review and have the potential to also be useful in the adult population. However, on the other hand, many measures used in the pediatric population are very specific to age and may not be appropriate in adult patients. Second, this study was a scoping review that broadly investigated the literature for studies exploring PF as the primary concept, therefore many studies that use PF COA to measure PF were not captured, in particular many therapeutic trials. Also, this study did not include a quality assessment tool to evaluate article quality. Lastly, many articles identified in this review did not provide specific time period information on when COAs were used to measure physical functioning, and most studies were descriptive in nature.

## Conclusion

This review suggests that ClinROs (ie KPS, ECOG, and FIM) are the most commonly used tools in neuro-oncology and that future studies should aim to standardize other COA measures (ClinRO, PRO, and PerfO) in the PBT population and specifically focus on standardizing objective measures including PerfO measures. Based on a literature search, currently there are no validated or standardized PerfO measures specifically for evaluating PF in brain tumor patients. Given that walking is the most common activity performed by an individual for maintaining independence measures testing walking capacity, speed, mobility, gait, and balance should be emphasized for validation in future studies.

## Supplementary material

Supplementary material is available online at *Neuro-Oncology Practice* (https://academic.oup.com/nop/).

npaf036_suppl_Supplementary_Materials
