# Supplementary material for: Measures of physical functioning in adults with brain tumor associated with functional outcomes: A scoping review
Source: Neurooncol Pract. 2025 Mar 26;12(4):571–84. doi: 10.1093/nop/npaf036 (PMC12349772; doi:10.1093/nop/npaf036)
Supplement: npaf036_suppl_Supplementary_Materials [file npaf036_suppl_supplementary_materials.docx]

**Supplementary Table and Figure Legends**

Supplemental Table 1 Preferred Reporting Items for Systematic Reviews and Meta-Analyses Extension for Scoping Reviews (PRISMA-ScR) Checklist

| **SECTION** | **ITEM** | **PRISMA-ScR CHECKLIST ITEM** | **REPORTED ON PAGE #** |
| --- | --- | --- | --- |
| **TITLE** | | | |
| Title | 1 | Identify the report as a scoping review. | 1 |
| **ABSTRACT** | | | |
| Structured summary | 2 | Provide a structured summary that includes (as applicable): background, objectives, eligibility criteria, sources of evidence, charting methods, results, and conclusions that relate to the review questions and objectives. | 1-2 |
| **INTRODUCTION** | | | |
| Rationale | 3 | Describe the rationale for the review in the context of what is already known. Explain why the review questions/objectives lend themselves to a scoping review approach. | 3-4 |
| Objectives | 4 | Provide an explicit statement of the questions and objectives being addressed with reference to their key elements (e.g., population or participants, concepts, and context) or other relevant key elements used to conceptualize the review questions and/or objectives. | 4 |
| **METHODS** | | | |
| Protocol and registration | 5 | Indicate whether a review protocol exists; state if and where it can be accessed (e.g., a Web address); and if available, provide registration information, including the registration number. | NA |
| Eligibility criteria | 6 | Specify characteristics of the sources of evidence used as eligibility criteria (e.g., years considered, language, and publication status), and provide a rationale. | 5 |
| Information sources* | 7 | Describe all information sources in the search (e.g., databases with dates of coverage and contact with authors to identify additional sources), as well as the date the most recent search was executed. | 5-6 |
| Search | 8 | Present the full electronic search strategy for at least 1 database, including any limits used, such that it could be repeated. | 5 & Supplemental Table 2 |
| Selection of sources of evidence† | 9 | State the process for selecting sources of evidence (i.e., screening and eligibility) included in the scoping review. | 6 |
| Data charting process‡ | 10 | Describe the methods of charting data from the included sources of evidence (e.g., calibrated forms or forms that have been tested by the team before their use, and whether data charting was done independently or in duplicate) and any processes for obtaining and confirming data from investigators. | 6-7 |
| Data items | 11 | List and define all variables for which data were sought and any assumptions and simplifications made. | 6-7 |
| Critical appraisal of individual sources of evidence§ | 12 | If done, provide a rationale for conducting a critical appraisal of included sources of evidence; describe the methods used and how this information was used in any data synthesis (if appropriate). | N/A |
| Synthesis of results | 13 | Describe the methods of handling and summarizing the data that were charted. | 7-8 |
| **RESULTS** | | | |
| Selection of sources of evidence | 14 | Give numbers of sources of evidence screened, assessed for eligibility, and included in the review, with reasons for exclusions at each stage, ideally using a flow diagram. | 9 |
| Characteristics of sources of evidence | 15 | For each source of evidence, present characteristics for which data were charted and provide the citations. | 9-16 |
| Critical appraisal within sources of evidence | 16 | If done, present data on critical appraisal of included sources of evidence (see item 12). | N/A |
| Results of individual sources of evidence | 17 | For each included source of evidence, present the relevant data that were charted that relate to the review questions and objectives. | 11-16 |
| Synthesis of results | 18 | Summarize and/or present the charting results as they relate to the review questions and objectives. | 11-16 |
| **DISCUSSION** | | | |
| Summary of evidence | 19 | Summarize the main results (including an overview of concepts, themes, and types of evidence available), link to the review questions and objectives, and consider the relevance to key groups. | 16-24 |
| Limitations | 20 | Discuss the limitations of the scoping review process. | 25 |
| Conclusions | 21 | Provide a general interpretation of the results with respect to the review questions and objectives, as well as potential implications and/or next steps. | 25 |
| **FUNDING** | | | |
| Funding | 22 | Describe sources of funding for the included sources of evidence, as well as sources of funding for the scoping review. Describe the role of the funders of the scoping review. | 26 |

JBI = Joanna Briggs Institute; PRISMA-ScR = Preferred Reporting Items for Systematic reviews and Meta-Analyses extension for Scoping Reviews.

* Where *sources of evidence* (see second footnote) are compiled from, such as bibliographic databases, social media platforms, and Web sites.

† A more inclusive/heterogeneous term used to account for the different types of evidence or data sources (e.g., quantitative and/or qualitative research, expert opinion, and policy documents) that may be eligible in a scoping review as opposed to only studies. This is not to be confused with *information sources* (see first footnote).

‡ The frameworks by Arksey and O’Malley (6) and Levac and colleagues (7) and the JBI guidance (4, 5) refer to the process of data extraction in a scoping review as data charting*.*

§ The process of systematically examining research evidence to assess its validity, results, and relevance before using it to inform a decision. This term is used for items 12 and 19 instead of "risk of bias" (which is more applicable to systematic reviews of interventions) to include and acknowledge the various sources of evidence that may be used in a scoping review (e.g., quantitative and/or qualitative research, expert opinion, and policy document).

Supplemental Table 2 Full Search Strategy in Scientific Databases (PubMed, Web of Science [Clarivate], Cochrane Library [Cochrane Reviews])

| **Databases** | **Terms** | **Hits** | **Date** |
| --- | --- | --- | --- |
| PubMed | ("Brain neoplasms"[Mesh] OR "brain neoplasm*"[tiab] OR "brain cancer"[tiab] OR glioblastoma[Mesh] OR glioma[Mesh] OR glioma[tiab] OR glioblastoma[tiab] OR Oligodendroglioma[Mesh] OR Meningioma[Mesh] OR meningioma[tiab] OR ependymoma[Mesh] OR ependymoma[tiab]) AND ("hand strength"[Mesh] OR "hand grip strength"[tiab] OR "timed up and go test"[tiab] OR mobility[tiab] OR balance[tiab] OR "usual walking speed"[tiab] OR "walking test*"[tiab] OR "6 minute walking test"[tiab] OR "walking test"[tiab] OR "balance assessment*"[tiab] OR "stand test"[tiab] OR "chair rise performance"[tiab] OR "Karnofsky Performance Status"[Mesh] OR "Karnofsky Performance Scale"[tiab] OR "medical research council scale"[tiab] OR "Eastern cooperative oncology group scale"[tiab] OR "modified Ashworth scale"[tiab] OR "lower extremity motor score"[tiab] OR "MDASI interference"[tiab] OR "PROMIS physical functioning"[tiab] OR "physical functioning"[tiab] OR "exercise capacity"[tiab] OR "physical fitness"[tiab] OR "physical performance"[tiab] OR "physical limitation"[tiab] OR "physical disability"[tiab] OR "functional status"[tiab] OR "functional capacity"[tiab] OR "functional ability"[tiab] OR "physical functional status"[tiab] OR "physical capacity"[tiab] OR "Motor activity"[Mesh] OR "motor activity"[tiab] OR walking[Mesh] OR walking[tiab] OR gait[Mesh] OR gait[tiab] OR “stair climbing”[Mesh] OR “stair climbing”[tiab] OR “muscle strength”[Mesh] OR “muscle strength”[tiab] OR “postural balance”[Mesh] OR “postural balance”[tiab] OR “core stability”[tiab] OR “posture balance”[tiab] OR “core balance”[tiab] OR “mobility limitation”[Mesh] OR “mobility limitation”[tiab] OR “difficulty walking”[tiab]) AND (“independence” [tiab] OR “disability” [tiab] OR “activities of daily living”[Mesh] OR “activities of daily living”[tiab] OR “daily living activities”[tiab] OR “independent living”[tiab] OR “functional status”[Mesh] OR “functional status”[tiab] OR “functional independence”[tiab] OR “functional dependence”[tiab] OR “health status”[Mesh] OR “health status”[tiab]) NOT **(****"Animals"[Mesh] NOT ("Animals"[Mesh] AND** **"Humans"[Mesh]))** | 622 | 25 Jan 2024 |
| Web of Science (Clarivate) | TOPIC:("Brain neoplasms" OR "brain neoplasm*" OR "brain cancer" OR glioblastoma OR glioma OR glioma OR glioblastoma OR Oligodendroglioma OR Meningioma OR meningioma OR ependymoma OR ependymoma) AND TOPIC:("hand strength" OR "hand grip strength" OR "timed up and go test" OR mobility OR balance OR "usual walking speed" OR "walking test*" OR "6 minute walking test" OR "walking test" OR "balance assessment*" OR "stand test" OR "chair rise performance" OR "Karnofsky Performance Status" OR "Karnofsky Performance Scale" OR "medical research council scale" OR "Eastern cooperative oncology group scale" OR "modified Ashworth scale" OR "lower extremity motor score" OR "MDASI interference" OR "PROMIS physical functioning" OR "physical functioning" OR "exercise capacity" OR "physical fitness" OR "physical performance" OR "physical limitation" OR "physical disability" OR "functional status" OR "functional capacity" OR "functional ability" OR "physical functional status" OR "physical capacity" OR "Motor activity" OR "motor activity" OR walking OR walking OR gait OR gait OR "stair climbing" OR "stair climbing" OR "muscle strength" OR "muscle strength" OR "postural balance" OR "postural balance" OR "core stability" OR "posture balance" OR "core balance" OR "mobility limitation" OR "mobility limitation" OR "difficulty walking") AND TOPIC:(independence OR disability OR "activities of daily living" OR "activities of daily living" OR "daily living activities" OR "independent living" OR "functional status" OR "functional status" OR "functional independence" OR "functional dependence" OR "health status" OR "health status") | 367 | 25 Jan 2024 |
| Cochrane Library (Cochrane Reviews) | ([mh "Brain neoplasms"] OR ("brain" NEXT neoplasm*):ti,ab OR "brain cancer":ti,ab OR [mh glioblastoma] OR [mh glioma] OR glioma:ti,ab OR glioblastoma:ti,ab OR [mh Oligodendroglioma] OR [mh Meningioma] OR meningioma:ti,ab OR [mh ependymoma] OR ependymoma:ti,ab) AND ([mh "hand strength"] OR "hand grip strength":ti,ab OR "timed up and go test":ti,ab OR mobility:ti,ab OR balance:ti,ab OR "usual walking speed":ti,ab OR ("walking" NEXT test*):ti,ab OR "6 minute walking test":ti,ab OR "walking test":ti,ab OR ("balance" NEXT assessment*):ti,ab OR "stand test":ti,ab OR "chair rise performance":ti,ab OR [mh "Karnofsky Performance Status"] OR "Karnofsky Performance Scale":ti,ab OR "medical research council scale":ti,ab OR "Eastern cooperative oncology group scale":ti,ab OR "modified Ashworth scale":ti,ab OR "lower extremity motor score":ti,ab OR "MDASI interference":ti,ab OR "PROMIS physical functioning":ti,ab OR "physical functioning":ti,ab OR "exercise capacity":ti,ab OR "physical fitness":ti,ab OR "physical performance":ti,ab OR "physical limitation":ti,ab OR "physical disability":ti,ab OR "functional status":ti,ab OR "functional capacity":ti,ab OR "functional ability":ti,ab OR "physical functional status":ti,ab OR "physical capacity":ti,ab OR [mh "Motor activity"] OR "motor activity":ti,ab OR [mh walking] OR walking:ti,ab OR [mh gait] OR gait:ti,ab OR [mh "stair climbing"] OR "stair climbing":ti,ab OR [mh "muscle strength"] OR "muscle strength":ti,ab OR [mh "postural balance"] OR "postural balance":ti,ab OR "core stability":ti,ab OR "posture balance":ti,ab OR "core balance":ti,ab OR [mh "mobility limitation"] OR "mobility limitation":ti,ab OR "difficulty walking":ti,ab) AND (independence:ti,ab OR disability:ti,ab OR [mh "activities of daily living"] OR "activities of daily living":ti,ab OR "daily living activities":ti,ab OR "independent living":ti,ab OR [mh "functional status"] OR "functional status":ti,ab OR "functional independence":ti,ab OR "functional dependence":ti,ab OR [mh "health status"] OR "health status":ti,ab) | 104 | 25 Jan 2024 |

Supplemental Table 3 Summary of sample of articles identified (N = 49)

| **Ref *^a^* #** | **Article** | **Country-Continent** | **N** | **Age Range (yrs *^b^*)** | **Mean Age (yrs *^b^*)** | **Median Age (yrs *^b^*)** | **Sex, Female (*n*)** | **Sex, Male (*n*)** | **Race, Ethnicity** | **Tumor**  **Type(s)** | **Study**  **Design** | **Type of**  ***^c^* PF  *^d^* COA Measures** |
| --- | --- | --- | --- | --- | --- | --- | --- | --- | --- | --- | --- | --- |
| ^1^ | Ahmadipour et al., 2019 | DEU-EU | 565 | 18-84 | Not stated | 62.2 | 241 | 324 | Not Stated | Glioblastoma | Prospective, Longitudinal | ClinRO |
| ^2^ | Albuquerque et al., 2023 | BRA-SA | 48 | 20-57 | 35.09 | Not stated | 21 | 27 | Not Stated | Gliomas | Prospective, Longitudinal | ClinRO |
| ^3^ | Back et al., 2019 | AUS-OC | 146 | Not Stated | Not Stated | 43 | 49 | 97 | Not  Stated | Gliomas | Prospective, Longitudinal | ClinRO |
| ^4^ | Bartolo et al., 2012 | ITA-EU | 75 | Not stated | 64.6 | Not stated | 45 | 30 | Not Stated | Meningioma, Glioblastoma | Prospective Case-Control, Longitudinal | ClinRO,  PerfO |
| ^5^ | Bette et al., 2016 | DEU-EU | 251 | Not Stated | 63.1 | Not Stated | 100 | 151 | Not Stated | Glioblastoma | Retrospective, Longitudinal | ClinRO |
| ^6^ | Bilgin et al., 2014 | TUR-AS | 68 | 18-60+ | 47.34 | Not Stated | 24 | 44 | Not  Stated | Gliomas | Retrospective Case-Control, Longitudinal | ClinRO |
| ^7^ | Brazil et al., 1997 | GBR-EU | 107 | 23-77 | Not stated | 57 | 39 | 68 | Not  Stated | Gliomas | Prospective, Longitudinal | ClinRO |
| ^8^ | Capozzi et al., 2015 | CAN-NA | 24 | Not stated | 52.2 | Not stated | 7 | 17 | Not  Stated | Gliomas (Oligo-dendroglioma Glioblastoma, Astrocytoma, Oligoastro-  cytoma, Ependymoma) | Prospective, Longitudinal | PerfO |
| ^9^ | Chaichana et al., 2011 | USA-NA | 544 | Not stated | 52 | Not stated | 211 | 333 | Not  Stated | Glioblastoma | Retrospective, Longitudinal | ClinRO |
| ^10^ | Culos-Reed et al., 2017 | CAN-NA | 15 | Not Stated | 50.6 | Not Stated | 7 | 8 | Not  Stated | Gliomas | Prospective, Longitudinal | PerfO |
| ^11^ | Dulfikar et al., 2021 | AUS-OC | 33 | 29-72 | 49 | Not stated | 12 | 21 | Not  Stated | Gliomas (Astrocytoma, Oligo-  dendroglioma, Glioblastoma) | Prospective, Cross-sectional | PerfO |
| ^12^ | Dutta et al., 2009 | IND-AS | 150 | 18-50+ | 39.9 | 40 | 62 | 88 | Not  Stated | Pilocytic astrocytoma, Diffuse fibrillary astrocytoma, Anaplastic astrocytoma, Glioblastoma, Ependymoma, Meningioma, Craniopharyngioma, Pituitary tumor, Medullo-  blastoma, Others | Prospective, Cross-sectional | ClinRO |
| ^13^ | Filippidis et al., 2023 | USA-NA | 30 | 80-89 | Not stated | 82.5 | 20 | 10 | Not Stated | Meningioma | Retrospective, Cross-Sectional | ClinRO |
| ^14^ | Flechl et al., 2012 | AUT-EU | 17 | 24-71 | Not stated | 51 | 9 | 8 | Not  Stated | Glioblastoma, Grade 4 | Prospective, Cross-Sectional | ClinRO,  PerfO |
| ^15^ | Greenberg et al., 2006 | ISR-AS | 1660 | Not stated | 58.13 | Not stated | 562 | 1266 | Not  Stated | Meningioma, Gliomas | Retrospective Case-Control, Longitudinal | ClinRO |
| ^16^ | Hansen et al., 2021 | DNK-EU | 81 | Not stated | 56.05 | Not stated | 27 | 54 | Not Stated | Gliomas | Retrospective, Cross-Sectional | PerfO |
| ^17^ | Heiland et al., 2018 | DEU-EU | 436 | 65+ | Not stated | 73.4 | Not stated | Not stated | Not Stated | Glioblastoma | Retrospective, Longitudinal | ClinRO |
| ^18^ | Huang et al., 1998 | USA-NA | 126 | 24-80 | 60 | Not stated | 64 | 62 | White: 65 Non-white: 61  Hispanic: 1  Non-Hispanic: 9 | Not stated | Prospective Case-Control, Longitudinal | ClinRO |
| ^19^ | Huang et al., 2001 | USA-NA | 10 | 31-71 | 55.9 | Not stated | 4 | 6 | Black: 4 White: 5 Not stated: 1 | Oligo-  dendrglioma, Meningioma, Glioblastoma, Colloid Cyst, Pituitary Adenoma | Prospective, Longitudinal | ClinRO |
| ^20^ | Januel et al., 2015 | FRA-EU | 81 | Not stated | Not stated | 64 | 34 | 47 | Not Stated | Glioblastoma | Retrospective, Longitudinal | ClinRO |
| ^21^ | Jones et al., 2009 | USA-NA | 171 | 20-77 | 49 | Not stated | 55 | 116 | Not  Stated | Gliomas | Prospective, Cross-Sectional | ClinRO,  PerfO |
| ^22^ | Jones, Friedman et al., 2010 | USA-NA | 35 | 22-77 | 47 | Not stated | 14 | 21 | Not  Stated | Gliomas | Prospective, Cross-Sectional | ClinRO,  PerfO |
| ^23^ | Jones, Mourtzakis et al., 2010 | USA-NA | 35 | 22-77 | 47 | Not stated | 14 | 21 | Not  Stated | Glioma | Prospective, Longitudinal | ClinRO,  PerfO |
| ^24^ | Joubert et al., 2021 | FRA-EU | 37 | 80-93.1 | Not stated | 81.9 | 20 | 17 | Not Stated | Meningioma | Retrospective, Longitudinal | ClinRO |
| ^25^ | Khan et al., 2013 | AUS-OC | 106 | 20.8-77.28 | 51.3 | Not stated | 61 | 45 | Not  Stated | Not stated (primary brain tumor) | Prospective, Cross-Sectional | ClinRO |
| ^26^ | Khan et al., 2014 | AUS-OC | 106 | 21-77 | 51 | Not stated | 61 | 45 | Not Stated | Gliomas, low and high grade | Controlled Clinical Trial | ClinRO |
| ^27^ | Kim et al., 2012 | KOR-AS | 25 | Not stated | 40 | Not stated | 11 | 14 | Not Stated | Meningioma, Low Grade Glioma, Pituitary adenoma, Glioblastoma multiforme, anaplastic astrocytoma, pinealo-blastoma, malignant melanoma, ewing sarcoma, and Choriocarcinoma | Prospective, Longitudinal | ClinRO |
| ^28^ | Kim et al., 2018 | KOR-AS | 51 | 19-65+ | 51.41 | Not stated | 21 | 30 | Not Stated | Anaplastic astrocytoma, Glioblastoma | Prospective, Longitudinal | ClinRO |
| ^29^ | Kombos et al., 2009 | DEU-EU | 40 | 31-69 | 51 | Not stated | 12 | 28 | Not Stated | Gliomas | Prospective Case-Control, Longitudinal | ClinRO |
| ^30^ | Konglund et al., 2012 | NOR-EU | 54 | 60-84 | Not stated | 70 | 35 | 19 | Not Stated | Meningioma | Prospective, Longitudinal | ClinRO |
| ^31^ | Kosteniuk et al., 2018 | CAN-NA | 24 | 19-57 ~ | 38 | Not stated | 10 | 14 | Not Stated | Gliomas | Retrospective Case-Control, Longitudinal | ClinRO |
| ^32^ | Krajewski et al., 2022 | POL-EU | 205 | 19-83 | 45.1 | Not stated | 109 | 96 | Not Stated | Not stated | Prospective Case-Control, Longitudinal | ClinRO,  PerfO |
| ^33^ | Li et al., 2021 | CHN-AS | 470 | 65-79 | 68.79 | Not stated | 332 | 138 | Not Stated | Meningioma | Retrospective, Longitudinal | ClinRO |
| ^34^ | Mainio et al., 2005 | FIN-EU | 77 | Not stated | 47.2 | Not stated | 47 | 30 | Not Stated | Meningioma, Glioma, Vestibular Schwannoma, Pituitary Adenoma, Hemangioperi-cytoma, Malignant Lymphoma, Craniopharyngioma, and Undefined. | Prospective, Longitudinal | ClinRO |
| ^35^ | Mainio et al., 2006 | FIN-EU | 77 | Not stated | 47.2 | Not stated | 47 | 30 | Not Stated | Glioma, meningioma, acoustic neurinoma, pituitary adenoma | Prospective, Longitudinal | ClinRO |
| ^36^ | Maschio et al., 2017 | ITA-EU | 25 | 28-70 | 41.9 | 48 | 7 | 18 | Not Stated | Astrocytoma, Glioblastoma, Oligo-  dendroglioma, Anaplastic Oligo-  astrocytoma, Anaplastic Oligo-dendroglioma, Anaplastic Astrocytoma | Prospective, Longitudinal | ClinRO |
| ^37^ | Nakajima et al., 2019 | JPN-AS | 60 | Not stated | 56 | Not Stated | 21 | 39 | Not Stated | Glioblastoma | Retrospective, Longitudinal | ClinRO |
| ^38^ | Nakajima et al., 2023 | JPN-AS | 47 | 32-85 | 66.1 | 66 | 21 | 26 | Note Stated | Glioblastoma | Retrospective, Longitudinal | ClinRO |
| ^39^ | Ness et al., 2010 | USA-NA | 156 | 18.4-58 | Not stated | 22 | 72 | 84 | White: 134  Non-White: 22 | Astrocytic, Medullo-  blastoma /Ependymoma, Other | Prospective Case-Control, Cross-Sectional | PerfO,  PRO |
| ^40^ | Rahal et al., 2022 | DEU-EU | 39 | Not stated | Not stated | 56.1 | 13 | 26 | Not  Stated | Glioblastoma | Retrospective, Longitudinal | ClinRO |
| ^41^ | Roberts et al., 2014 | USA-NA | 412 | Not Stated | 60.8 | 62 | 165 | 247 | Not  Stated | Glioblastoma | Retrospective, Longitudinal | ClinRO |
| ^42^ | Sacko et al., 2015 | FRA-EU | 84 | Not stated | Not stated | 61 | 33 | 51 | Not  Stated | Glioblastoma | Retrospective, Longitudinal | ClinRO |
| ^43^ | Sass et al., 2022 | USA-NA | 579 | 18-85 | 50 | 50 | 243 | 336 | Asian/  Native Hawaiian/ Islander: 60  Black: 76  White: 866  Missing/ Other: 50  Hispanic: 106 | Astrocytoma, oligo-dendroglioma, ependymoma, other, no tissue diagnosis | Prospective, Cross-Sectional | ClinRO |
| ^44^ | Schiavolin et al., 2022 | ITA-EU | 149 | Not Stated | 52.5 | Not Stated | 92 | 57 | Not  Stated | Gliomas, Meningioma | Prospective, Longitudinal | ClinRO,  PRO |
| ^45^ | Spychka et al., 2019 | CAN-NA | 30 | Not Stated | 55.8 | Not Stated | 16 | 14 | Not  Stated | Gliomas (Glioblastoma, Astrocytoma, Oligo-  dendroglioma, Meningioma, Oligoastro-cytoma, other Gliomas) | Retrospective, Cross-Sectional | PerfO,  PRO |
| ^46^ | Tabouret et al., 2016 | FRA-EU | 71 | 19-85 | 56 | Not Stated | Not stated | Not stated | Not  Stated | Gliomas (Anaplastic Glioma, Glioblastoma) Primary Cerebral Lymphoma, others | Retrospective, Longitudinal | ClinRO |
| ^47^ | Tankumpuan et al., 2015 | THA-AS | 88 | 18-72 | 45.18 | Not Stated | 66 | 22 | Not  Stated | Meningioma, Pituitary Adenoma, Glioblastoma, others | Prospective, Cross-Sectional | PRO |
| ^48^ | Varedi et al., 2021 | USA-NA | 329 | 18-53 | 27.5 | Not stated | 138 | 191 | Black: 51  White: 273  Other: 5  Hispanic: 3  Non-Hispanic: 326 | Astroglia, Medullo-  blastoma, Ependymoma, Cranio-  pharyngioma, Germ cell, others | Prospective, Cross-Sectional | PerfO |
| ^49^ | Zhang et al., 2017 | CH-AS | 104 | Not Stated | 40 | Not Stated | 66 | 38 | Not  Stated | Gliomas | Retrospective, Longitudinal | ClinRO |

*^a^* Ref (Reference), *^b^* Yrs (Years) *^c^* PF (Physical Functioning), *^d^* COA (Clinical Outcome Assessment)

AS (Asia), AUS (Australia), AUT (Austria), BRA (Brazil), CAN (Canada), CHN (China), DEU (Germany), DNK (Denmark), EU (Europe), FIN (Finland), FRA (France), GBR (Great Britian), IND (India), ISR (Israel), ITA (Italy), JPN (Japan), KOR (Korea), NA (North America), NOR (Norway), OC (Oceania), POL (Poland), SA (South America), THA (Thailand), TUR (Turkey), USA (United States),

ClinRO (Clinician-Reported Outcome), PerfO (Performance Outcome), PRO (Patient-Reported Outcome)

Supplemental Table 4 Physical functioning domains evaluated for each study

| **Ref #** | **Article** | **Activities of daily living**  **(n = 45)** | **Ambulation (n = 10)** | **Balance (n = 6)** | **Cognition (n = 10)** | **Dexterity (n = 2)** | **Mobility (n = 14)** | **Muscle Strength (n = 10)** | **Oxygen Consuption Capacity**  **(n = 2)** |
| --- | --- | --- | --- | --- | --- | --- | --- | --- | --- |
| ^1^ | Ahmadipour et al., 2019 | X |  |  |  |  |  |  |  |
| ^2^ | Albuquerque et al., 2023 | X |  |  |  |  |  |  |  |
| ^3^ | Back et al., 2019 | X | X |  |  |  |  |  |  |
| ^4^ | Bartolo et al., 2012 | X | X | X | X |  | X |  |  |
| ^5^ | Bette et al., 2016 | X |  |  |  |  |  |  |  |
| ^6^ | Bilgin et al., 2014 | X |  |  |  |  |  |  |  |
| ^7^ | Brazil et al., 1997 | X |  |  |  |  |  |  |  |
| ^8^ | Capozzi et al., 2015 |  |  |  |  |  | X | X |  |
| ^9^ | Chaichana et al., 2011 | X |  |  |  |  |  |  |  |
| ^10^ | Culos-Reed et al., 2017 |  |  |  |  |  |  | X | X |
| ^11^ | Dulfikar et al., 2021 |  | X | X |  |  | X |  |  |
| ^12^ | Dutta et al., 2009 | X |  |  | X |  | X |  |  |
| ^13^ | Filippidis et al., 2023 | X |  |  |  |  |  |  |  |
| ^14^ | Flechl et al., 2012 | X | X | X |  | X | X |  |  |
| ^15^ | Greenberg et al., 2006 | X |  |  | X |  | X |  |  |
| ^16^ | Hansen et al., 2021 | X |  |  |  |  | X | X | X |
| ^17^ | Heiland et al., 2018 | X | X | X | X |  |  | X |  |
| ^18^ | Huang et al., 1998 | X |  |  | X |  | X |  |  |
| ^19^ | Huang et al., 2001 | X |  |  | X |  | X |  |  |
| ^20^ | Januel et al., 2015 | X |  |  |  |  |  |  |  |
| ^21^ | Jones et al., 2009 | X | X |  |  |  | X |  |  |
| ^22^ | Jones, Friedman et al., 2010 | X |  |  |  |  |  | X | X |
| ^23^ | Jones, Mourtzakis et al., 2010 | X |  |  |  |  |  | X | X |
| ^24^ | Joubert et al., 2021 | X |  |  |  |  |  |  |  |
| ^25^ | Khan et al., 2013 | X |  |  |  |  | X |  |  |
| ^26^ | Khan et al., 2014 | X |  |  | X |  | X |  |  |
| ^27^ | Kim et al., 2012 | X |  |  |  | X | X | X |  |
| ^28^ | Kim et al., 2018 | X |  |  |  |  |  |  |  |
| ^29^ | Kombos et al., 2009 | X |  |  |  |  |  |  |  |
| ^30^ | Konglund et al., 2012 | X |  |  |  |  |  |  |  |
| ^31^ | Kosteniuk et al., 2018 | X |  |  |  |  |  |  |  |
| ^32^ | Krajewski et al., 2022 | X | X |  |  |  |  |  |  |
| ^33^ | Li et al., 2021 | X |  |  |  |  |  |  |  |
| ^34^ | Mainio et al., 2005 | X |  |  |  |  |  |  |  |
| ^35^ | Mainio et al., 2006 | X |  |  |  |  |  |  |  |
| ^36^ | Maschio et al., 2017 | X |  |  |  |  |  |  |  |
| ^37^ | Nakajima et al., 2019 | X |  |  |  |  |  |  |  |
| ^38^ | Nakajima et al., 2023 | X |  |  |  |  | X | X |  |
| ^39^ | Ness et al., 2010 | X |  |  |  |  | X | X | X |
| ^40^ | Rahal et al., 2022 | X |  |  |  |  |  |  |  |
| ^41^ | Roberts et al., 2014 | X |  |  | X |  | X |  |  |
| ^42^ | Sacko et al., 2015 | X |  |  |  |  |  |  |  |
| ^43^ | Sass et al., 2022 | X |  |  |  |  |  |  |  |
| ^44^ | Schiavolin et al., 2022 | X |  |  | X |  | X |  |  |
| ^45^ | Spychka et al., 2019 | X | X | X |  |  | X | X |  |
| ^46^ | Tabouret et al., 2016 | X |  |  |  |  |  |  |  |
| ^47^ | Tankumpuan et al., 2015 | X | X |  |  |  | X |  |  |
| ^48^ | Varedi et al., 2021 | X | X |  | X |  | X | X |  |
| ^49^ | Zhang et al., 2017 | X |  |  |  |  |  |  |  |

Supplemental Table 5 ClinRO COA Measures Identified

| **Measure** | **Physical Functioning Domain(s) Measured** | **Description** |
| --- | --- | --- |
| Barthal Index (BI) | Activities of daily living | Measures ten common activities of daily living including basic mobility (i.e., feeding, bathing, grooming, dressing, bowel, bladder, toilet use, transfers bed-to-chair-and-back, mobility on level surfaces, stair negotiation) in the last 24 to 48 hours. Scores range from 0 to 100, where lower scores indicate less independence^50,51^. *The BI is a standard and widely used measure of activties of daily living in patients after a stroke*^52^. |
| Brunnstrom Recovery Stage Index (BRSI) | Mobility | Measures motor functioning ability which consists of three items for the arms, hands, and lower limbs, testing for stage 1 (flaccidity), stage 2 (spasticity), stage 3 (co-movement), stage 4 (partial dissociative movement), stage 5 (dissociative movement), or stage 6 (normal movement). Scores range from stage 1 to 6, where stage 1 indicates flacidity (undable to move muscles, and feeling limp and floppy) and stage 6 normal movement (control over movements and almost fully return to typical function, involuntary muscle tightening diappears, and movements are coordinated)^53,54^. |
| Disability Rating Scale (DRS) | Activities of daily living, cognition | Measures patients cognition and activities of daily living within four categories 1) arousal, awareness, and reponse to command, 2) cognitive ability for self-care, 3) dependence on others, 4) psychosocial adaptability. Each category is scored 0 to 3 or 0 to 5, with total scores range from 0 to 30. Higher scores indicate more disability and a score of 30 indicating death^55^. *The DRS a common outcome measure used in mild to severe traumatic brain injury patients and is an alternative to the Glasgow Outcome Scale or the Extended Glasgow Outcome Scale*^56^. |
| Eastern Cooperative Oncology Group (ECOG) | Activities of dailly living, Ambulation | Measures abilities to ambulate and complete activities of daily living in oncology patients receiving *standard cancer treatments or patients being considered for clincal trials*. Scores range from grade 0 to 5, where grade 0 (indicates fully active and able to carry on all pre-disease performance without restriction), grade 1(restricted in physically strenuous activity but ambulatory and able to do light sedentary work), grade 2 (ambulatory about or more than 50% of waking hours and able to do all selfcare but unable to carry out work activities), grade 3 (limited selfcare, confined to bed or chair 50% of waking hours), grade 4(cannot carry out any self care, totally confined to bed or chair), grade 5 (death)^57,58^. This measure is also sometimes referred to as the World Health Organization Performance Status (WHO PS)^59^. |
| Functional Activity Measure System (FIM-FAM) | Activities of daily living, Cognition, Mobility | Measures activities of daily living and encompasses a few cognition items (i.e., self care, sphincter control, mobility, locomotion, communication, psychosocial adjustment, cognitive function) assessed within 72 hours of admission to rehabiliation, and within 72 hours prior to the completion of rehabilitation. Scores range from 30 to 210 where high scores indicate completion of tasks independently and safely^12,60^. |
| Functional Ambulation Category/  Massachusetts General Hospital Functional Ambulation Classification (FAC) | Ambulation | Measures ambulation ability rehardless of a personal assistive device is used. Scoring ranges from 0 to 5, where 5 ambulator independent (indicates a patient who can walk everywhere independently, including stairs), 4 ambulator independent level surface only (indicates a patient who can ambulate independently on level surface but requires supervision to negotiate), 3 ambulator dependent on supervision (indicates a patient who can ambulate on level surface without manual contact of another person but requires standby guarding of one person either for safety or verbal cueing), 2 ambulator dependent on physical assistance (indicates a patient who requires intermittent or continuous light touch to assist balance or coordination, 1 ambulator, dependent on physical assistance (indicates a patient who requires continuous manual contact to support body weight as well as to maintain balance or to assist coordination^61,62^. |
| Functional Independence Measure (FIM) | Activities of daily living, Cognition | Measures activities of daily living, self care, sphincter control, transfer, locomotion, communication, social cognition, cognition, and mobility assessed within 72 hours of admission to rehabilitation, and within 72 hours prior to the completion of rehabilitation. Scores range from 18 to 126 where high scores indicate more independent the patient is in performing tasks^63,64^. *The FIM is validated broadly in patients undergoing rehabilitation*^65^ *and has been commonly used to measure activties of daily living in the neuro-oncology population as well as in patients following a stroke*^66^ *or traumatic brain injury*^65^. |
| Hauser Ambulation Index (AI) | Ambulation, Mobility | Measures mobility by evaluating the time and degree of assistance required to walk 25 feet. Scores from 0 to 9, where grade 0 (indicates fully active and asymptomatic), grade 1 (walks normally, but reports fatigue that inteferes with athletic or other demanding activities), grade 2 (abnormal gait or episodic imbalance, gait disorder noticed by family and friends, able to walk 25 feet [8 meters] in 10 seconds or less), grade 3 (walks independently, able to walk 25 feet in 20 seconds or less), grade 4 (requires unilateral support [cane/single crutch] to walk, walks 25 feet in 20 seconds or less), grade 5 (requires bilateral support [cane/single crutch] to walk, walks 25 feet in 20 seconds or less; or requires unilateral support but needs more than 20 seconds to walk 25 feet), grade 6 (requires biolateral support and more than 20 seconds to walk 25 feet, may use wheelchair on occasion), grade 7 (walking limited to several steps with bilateral support, unable to walk 25 feet; may use a wheelchair for most activities, grade 8 (restricted to wheelchair; able to transfer self independently) and stage 9 (restricted to wheelchair, unable to transfer self independently^67^. |
| International Classification of Functioning (ICF) | Activities of daily living | Is a framework that measures functioning and disability within a standard enviorment (measures level of capacity) and in their usual environment (measures level of performance) in the context of an individuals health status or condidtion. It focuses on three components 1) body functions and structures, 2) activities and participation, 3) personal and environmental factors. The scoring measures impairments, limitations and restrictions and ranges from 0 to 100%, where 0 to 4% indicates no problem, 5 to 24% indicates mild problem, 25 to 49% indicates moderate problem, 50 to 95% indicates severe problem, and 96 to 100% indicates complete problem^68^. |
| Karnofsky Performance Status Scale (KPS) | Activities of daily living | Measures a patient’s ability to perform daily tasks and functional status. The scores range from 0 to 100%, where 100% (indicates normal function, no complaints, no evidence of disease), 90% (able to carry on normal activity, minor signs or symptoms of disease), 80% (normal activity with effort, some signs or symptoms of disease), 70% (Cares for self, unable to carry on normal activity or to do active work), 60% (requires occasional assistance, but is able to care for most of his needs), 50% (requires considerable assistance and frequent medical care), 40% (disabled requires special care and assistance, in bed more than 50% of the time), 30% (severely disabled, hospitalization is indicated although death not imminent, almost competely bedfast), 20% (hospitalization necessary, very sick, active supportive treatment necessary, totally bedfast and requiring extensive nursing care by professionals and/or family), 10% (moribund, fatal processes progressing rapidly, comatose or barely arousable), 0% (death)^69,70^. *A standard tool used for cancer patients and most commonly used in brain tumor patients*^71^. |
| Modified Rankin Scale (mRS) | Activities of daily living | Measures dependence in activites of daily living within the last 3 months following hospital or rehabilitation discharge. Scores range from 0 to 8, where 0 (indicates no symptoms), 1 (no significant disability, able to perform all usual activities despite some symptoms), 2 (slight disability, able to look after own affairs without assistance but unable to perform all previous activities, 3 (moderate disability, requires some help but able to walk without assistance), 4 (moderately severe disability, unable to walk without assistance or attend to own bodily needs without assistance), 5 (severe disability, bedridden, incontinent, and requires constant nursing care and attention, 6 (death)^72,73^. *The mRS a standard measure for assessing activties of daily living in patient’s after a stroke*^74^ |
| Motricity Index (MI) | Muscle Strength, Mobility, Dexterity | Measures upper extremities for strength including shoulder abduction, elbow flexion, pinch grip and lower extremities for hip flexion, knee extension, dorsiflexion. Scores range from 0 to 33, where when assessing grip 0 (indicates no movement), 11 (beginnings of prehension), 19 (able to grip, but not hold it against gravity examiner may need to lift the wrist), 22 (able to grip and hold the cube against gravity), 26 (able to grip and hold the cube against a weak pull, but weaker than the other side, 33 (normal power). Scoring for all other movements except grip includes 0 (no movement), 9 (palpable contraction in muscle, but no movement), 14 (visible movement, but not full range and not against gravity), 19 (Full range of movement against gravity, but not resistance) 25 (full movement against gravity but weaker than the other side), 33 (normal power)^75-77^. |
| Manual Muscle Test (MMT) | Muscle strength | Measures the function of muscle strength and evaluates weakness. The MMT can be used on different muscle groups including upper and lower extremities. Scores range from 0 to 5 where 1 indciates flicker of movement, 2 indicates full range actively with gravity counterbalanced, 3 indicates full range actively against gravity, 4 indicates full range actively against some resistance, and 5 indicates full range actively against strong resistance^78,79^. |
| Neurological Performance Score (NPS) | Activities of daily living | Measures neurological status including workability, functional ability. Scores range from grade 0-4, where grade 0 (indicates no neurological deficit), 1 (some neurological deficit but function adequate for useful work, 2 (neurological deficit causing moderate functional impairment), 3 (neurological deficit causing major functional impairment, and 4 (no useful function – inability to make conscious responses)^80^. |

Supplemental Table 6 PRO COA Measures Identified

| **Measure** | **Physical Functioning Domain(s) Measured** | **Description** |
| --- | --- | --- |
| Duke Activity Status Index (DASI) | Activities of daily living, Mobility, Oxygen consuption capacity | Measures a patient’s functional capacity including activities of daily living, mobility, workability, social and recreation activities. It also can be used as a surrogate measure for a patient’s peak oxygen uptake (cardiopulmonary fitness). Scores range from 0 to 58.2, where higher scores indicate a higher functional capacity^81,82^. |
| Functional Status Index (FSI) | Activities of daily living | Measures a patient’s functional ability including activities of daily living, physical activity, workability, and social activties. Functional ability is evalued under 3 domains, degree of dependence, degree of difficulty, and amount of pain experienced in the last month. Scores range from 0 to 100, where lower scores indicate worse function^83^. |
| Rosow-Breslau Physical Activity Self-Assessment (RSB) | Activities of daily living, Mobility | Measures physical performance status including activities of daily living (ability to do heavy work around the house) and mobility (walking without assistance and walking up and downstairs without assistance). Scores range from 0 to 3, where 0 indicates lowest functioning and 3 indicates highest functioning^84^. |
| Sickness Impact Profile (SIP) | Activities of daily living, Ambulation, Mobility | Measures a patient’s physical and psychosocial functioning which includes 12 cateogries (sleep/rest, eating, work, home management, recreation, ambulation, mobility, body care interaction, alertness, emotion, and communication. Scores range from 0 to100, where lower scores indicate better physical and psychosocial functioning^85^. *The SIP is used frequently to measure physical functioning in patients participating in stroke research*^86^. |
| World Health Organization Disability Assessment Schedule (WHODAS) | Activities of daily living, Mobility, Cognition | Measures disability and was developed from a large pool of items from the International Classification of Functioning (ICF). Patients are asked to indicate how difficult it is to performe a series of given tasks and activies taking into account the need for support (including from a caregiver) or assesive devices. For any item rated rater than none a follow up question is used to identify frquency and degree of difficulty experienced in the past 30 days. Each item total has different ranges and is dependent on the WHODAS version^87,88^. |

Supplemental Table 7 PerfO COA Measures Identified

| **Measure** | **Physical Functioning Domain(s) Measured** | **Description** |
| --- | --- | --- |
| 1-Repetition Maximum Test (1-RMT) | Muscle Strength | Measures isotonic muscle strength based on the maximal weight a patient can lift with one repeition. A variety of equipment can be used to assess strength of different muscles. The maximum weight lifted is recorded after performing 3-8 repetition maximum^16,89^. |
| 6-Minute Walking Test (6-MWT) | Mobility, Ambulation | Measures walking capacity, aerobic endurance, and community walking prediction in patients. *Commonly used in patients during stroke recovery*^90^. Scores are based on the distance a patient walks in 6 minutes^91^. |
| 9-Hole Peg Test  (9-HPT) | Dexterity | Measures finger dexterity based on a patient’s timed ability to take pegs from a container and place them into holes on a board and then remove the pegs from the holes and place them back into the container^92,93^. *The 9-HPT is known to be reliable, valid and sensitive to change in patients after a stroke*^94^. |
| 10-Meter Walking Test (10-TMW) | Mobility, Ambulation, Balance | Measures functional mobility, gait, and vestibular function based on walking speed in meters per second for 10 meters. The patient is instructed to walk a total of 14 meters, which includes a 2-meter initiation and termination phase. Total time needed to ambulate 10 meters is recorded regardless if assistive device is needed^95,96^. *The 10TMW measures has been validated in spinal cord injury patients*^97^ *and Stroke patients*^90^. |
| 10-Point Gait Index (10-PGI) | Ambulation | Measures gait efficiency and is based on 10 different tasks graded in 1-point increments including 1) impossible to achieve an upright vertical position, 2) possible to stand with the assistance of the therapist who secures the knees, hips, and trunk, 3) independent standing, the possibility of supporting with equipment, 4) gait while learning to walk with a therapist, no the possibility of practical use, 5) gait with the assistance of another person, but only within a room, accessing the wheelchair toilet, 6) gait with the assistance of another person, distance of several dozen meters (walking in the hospital corridor), 7) independent gait with a walking frame, 8) independent gait with crutches or walking stick, 9) incorrect independent gait, 10) correct independent gait^32^. |
| 30-Second Sit-to-Stand Test (30-CST) | Mobility, Muscle Strength | Measures functional lower extremity strength, based on the patient’s ability to stand in 30 seconds The patient is instructed to complete as many full stands as possible within 30 seconds. The score is the total number os stand within 30 seconds^98^. |
| Assessment of Motor and Process Skills (AMPS) | Activities of daily living | Measures the quality of a patient’s activities of daily living based on effort, efficiency, safety, and independence of 16 motor (body position, obtaining and holding an object, moving self, and objects) and 20 processing skills (apply knowledge, temporal organization, organizing space and objects, adapting performance). Each item ranges from 1 to 4, where higher scores indicate that the patient performs the task without increased effort, decreased efficiency, or lack of safety^99,100^. |
| Astrand-Rhyming Cycle Ergometer Test/ Maximum or Peak Volume Oxygen (VO_2 max/peak_) | Oxygen consuption capacity | Measures aerobic capacity and VO_2_ max (maximum amount of oxygen the body can absorb and use for exercise) with a submaximal test using a cycle ergometer. The patient pedals for 6 minutes at a load chosen by age and sex to induce a heart rate of 125-170 beats per minute. Scoring is determined using a nomogram based on VO_2_ max and steady-state pulse rate acheived during the 6-minute testie. This submaximal test was designed as an alternative for maximal tests for older individuals or people with cardiovascular diseases ^101^. |
| Berg Balance Scale (BBS) | Balance, Mobility | Measures balance and mobility abilities based on 14 tasks including 1) move from a sitting to a standing position, 2) stand up unsupported, 3) sit unsupported, 4) move from a standing to a sitting position, 5) transfer from one chair to another, 6) stand up with eyes closed, 7) stand with feet together, 8) reach forward with an outstretched arm, 9) pick an object up off the floor, 10) turning to look behind, 11) turning 360 degrees, 12) placing alternate foot on a stool, 13) stand unsupported with one foot directly in front of the other, 14) stand on one leg for as long as the patient can. Each item is scored from 0 to 4 and determined based on ability to perform each task. Total scores range from 0 to 56, where a score lower scores indicate higher risk of falls in a patient^102-104^. *The BBS is reliable and valid measure in rehabilitation after a strok*e^105,106^. |
| Cycling Exercise Test/Maximum or Peak Volume Oxygen (VO_2 max/peak_) | Oxygen consuption capacity | Measures aerobic capacity by assessing VO_2_ _max/peak_ (maximum amount of oxygen the body can absorb and use for exercise) with an incremental cycling test performed on an electrically braked cycle ergometer with 12-lead ECG. Patients warm up for 3 minutes and their metabolic data is collected. The patient begins cycling at 20 workload per minute and workload increases 5-20 per minute based on warmup metabolic data until exhaustion or symptomatic indication. VO_2 peak_ is determined by breath-by-breath expired gas analysis^107^. |
| Dynamic Strength Leg Press (DSLP) | Muscle strength | Measures functional dynamic muscle strength of the hip and knee flexors with leg press resistence equipment. Patients are asked to make 3 to 8 repetitions with of a maximal load. The maximal load weight completed with proper form for at least one repetition is measured^16^. |
| Hand Grip Strength (HGS) | Muscle strength | Measures muscular strength and maximum force or tension generated by a patient’s forearm muscles. Hand grip strength is also used to measure upper body strength and overall strength. Usually hand grip strength is measured using a handheld dynamometer. To assess hand grip strength the patient is 1) seated with shoulder adducted, 2) elbow is flexed at 90 degrees, forearm and wrist neutral, 3) the arm is not supported by examiner or armrest and the dynamometer is presented vertically, 4) the patient is instructed to squeeze as hard as possible, 5) maximum grip is the mean of 3 trials. The force is measured in kilograms, pounds, millilitres of mercury or in Newtons^108-110^. |
| Muscle Strength of Right Quadriceps, Plantar | Muscle strength | Isokinetic testing that consists of 3 sequential voluntary maximal contractions at an angular velocity of 90 degrees per second. Maximal isokinetic strength is the highest peak torque achieved during the 3 contractions^22^. |
| Physical Performance Test (PPT) | Activities of daily living, Mobility, Cognition | Measures different domains of physical function including activities of daily living, mobility, and cognition. Domains are measured using 9 or 7 timed tasks. Two versions of the PPT exist (9-item scale and 7-item scale). Scores range from 0 to 36 for the 9-item scale and 0 to 28 for the 7-item scale, where a higher score indicates better physical performance^111^. |
| Short Physical Performance Battery (SPPB) | Mobility, Balance, Ambulation | Measures lower extremity physical performance status using three timed tasks including standing balance (the patient stands with their feet in 3 different positions: side-by-side, semi-tandem, and tandem), 4-meter and 3-meter walking speed (the patient walks at a usual pace), and chair standing speed (the patient rises from a chair 5 times). The SPPB has also been shown to be a proxy measure for biological age in older individuals. Scores range from 0 to 12, where 0 to 6 indicates poor performance, 7 to 9 indicates moderate performance, and 10-12 indicates good performance^112,113^. |
| Sit-and-Reach Test (SRT) | Flexibility | Assesses gait and fall risk based on the extension of hamstrings and lower back. The patient sits on the floor with their legs extended and slowly reaches their arms forward as far as possible along a yardstick. The score is the furthest point the patient reached with their fingertips. Scores are split into percentiles according to normative values by age and gender and rated: well above average, below average, average, above average, and well above average. |
| Sitting Balance | Balance | Assesses static and dynamic balance in the sitting position, by evaluating the patient sitting with feet on the floor, back unsupported, and hands on the lap for 15 seconds. The evaluator then nudges anteriorly, posteriorly, and laterally (using ~5-10 foot pounds of force) while guarding the patient from falling with the free hand. Scores range from 1 to 4, where 4 (normal balance) indicates that the patient can perform the test without any physical assistance, 3 (good balance) patient is able to maintain a static position without difficulty but reqires assistance in righting reflex reponse from the hemiplegic side, 2 (fair balance) patient is able to maintain a static position without difficulty but requiring assistance in all righting reflex response tasks, 1 (poor balance) patient is unable to maintain a static position^114^. |
| Standing Balance | Balance | Assesses ability to stand, by evaluating the patient standing balance on a smooth floor wearing shoes without orthoses. The patient is instructed that three attempts will be preformed. Scores range from 0 to 4 where a 4 means that an individual is able to maintain a standing posture safely^115^. |
| Timed-Up-and-Go Test (TUG) | Mobility, Balance, Ambulation | Measures mobility, balance, ambulation and fall risk. A modified timed version of the ‘Get-Up and Go Test’, where a patient is observed and timed while rising from a arm chair, walks 3 meters, turns, walks back, and sits down again. Patient’s postural stability, gait, stride length, sway, and if assistive device is used are evaluated by the observer. Scoring is based on the timed task, where 10 seconds or less (indicates normal mobility), 11 to 20 seconds (indicate normal limits for frail elderly and disabled patients), 20 to 30 seconds (problems, may need assistance outside, and may be prone to falls, 30 seconds or more (limited mobility and an increased risk of falling)^116^. |
| Watt-Max Cycling | Oxygen consumption capacity | Measures aerobic capacity using the oxygen uptake with increasing watt (W) loads while on a mechanically braked cycle ergometer. The patient is allowed a 7-minute warmup at a sub-maximal load of 103 W for men and 69 W for women. The load is increased by 35 W for every 2 minutes until exhaustion. The maximal power output is recorded as the last complete load in W before exhaustion and VO_2_ _max_ is set as the highest value of oxygen uptake in the last 4 minutes of the test^117^. |

*Supplemental Table 7* Abbreviations for Measures Identified in this Review

1-RMT – Repetition Maximum Test

6-MWT – 6-Minute Walking Test

9-HPT – 9-Hole Peg Test

10-TMW – 10-Meter Walking Test

10-PGI – 10-Point Gait Index

30-CST – 30-Second Sit-To-Stand Test

AI – Hauser Ambulation Index

AMPS – Assessment of Motor and Process Skills

BBS – Berg Balance Scale

BI – Barthal Index

BRSI – Brunnstrom Recovery Stage Index

DASI – Duke Activity Status Index

DRS – Disability Rating Scale

DSLP – Dynamic Strength Leg Press

ECOG – Eastern Cooperative Oncology Group

FIM-FAM – Functional Activity Measure System

FAC – Functional Ambulation Category/Massachusetts General Hospital Functional Ambulation Classification

FIM – Functional Independence Measure

FSI – Functional Status Index

HGS – Hand Grip Strength

ICF – International Classification of Functioning

KPS – Karnofsky Performance Status Scale

mRS – Modified Rankin Scale

MI – Motricity Index

MMT – Manual Muscle Test

NPS – Neurological Performance Score

PPT – Physical Performance Test

RSB – Rosow-Breslau Physical Activity Self-Assessment

SIP – Sickness Impact Profile

SPPB – Short Physical Performance Battery

SRT – Sit-and-Reach Test

TUG – Timed-Up-and-Go Test

VO_2 max/peak_ – Astrand-Rhyming Cycle Ergometer Test/Cycling Exercise Test/Maximum or Peak Volume Oxygen

WHODAS – World Health Organization Disability Assessment Schedule

*References*

1. Ahmadipour Y, Kaur M, Pierscianek D, et al. Association of Surgical Resection, Disability, and Survival in Patients with Glioblastoma. *Journal of Neurological Surgery Part a-Central European Neurosurgery.* Jul 2019;80(4):262-268.

2. Albuquerque LAF, Macêdo LJM, Borges FS, et al. Awake Craniotomy for Diffuse Low Grade Gliomas in a Resource Limited Setting: Lessons Learned with a Consecutive Series of 51 Surgeries. *World Neurosurgery.* Sep 2023;177:E563-E579.

3. Back MF, Jayamanne D, Back E, et al. Reflecting on survivorship outcomes to aid initial decision making in patients treated for IDH-mutated anaplastic glioma. *Cancer.* Oct 2019;125(19):3457-3466.

4. Bartolo M, Zucchella C, Pace A, et al. Early rehabilitation after surgery improves functional outcome in inpatients with brain tumours. *Journal of Neuro-Oncology.* May 2012;107(3):537-544.

5. Bette S, Wiestler B, Kaesmacher J, et al. Infarct volume after glioblastoma surgery as an independent prognostic factor. *Oncotarget.* Sep 20 2016;7(38):61945-61954.

6. Bilgin S, Kose N, Karakaya J, Mut M. Traumatic brain injury shows better functional recovery than brain tumor: a rehabilitative perspective. *European Journal of Physical and Rehabilitation Medicine.* Feb 2014;50(1):17-23.

7. Brazil L, Thomas R, Laing R, et al. Verbally administered Barthel Index as functional assessment in brain tumour patients. *Journal of Neuro-Oncology.* Sep 1997;34(2):187-192.

8. Capozzi LC, Boldt KR, Easaw J, Bultz B, Culos-Reed SN. Evaluating a 12-week exercise program for brain cancer patients. *Psychooncology.* Mar 2016;25(3):354-358.

9. Chaichana KL, Halthore AN, Parker SL, et al. Factors involved in maintaining prolonged functional independence following supratentorial glioblastoma resection Clinical article. *Journal of Neurosurgery.* Mar 2011;114(3):604-612.

10. Nicole Culos-Reed S, Leach HJ, Capozzi LC, Easaw J, Eves N, Millet GY. Exercise preferences and associations between fitness parameters, physical activity, and quality of life in high-grade glioma patients. *Support Care Cancer.* Apr 2017;25(4):1237-1246.

11. Dulfikar A, Koh ES, Lwin Z, et al. Physical functional capacity of patients with glioma prior to adjuvant radiation: preliminary descriptive study. *Neuro-Oncology Practice.* Jun 2021;8(3):290-298.

12. Dutta D, Vanere P, Gupta T, Munshi A, Jalali R. Factors influencing activities of daily living using FIM–FAM scoring system before starting adjuvant treatment in patients with brain tumors: results from a prospective study. *Journal of Neuro-Oncology.* 2009/08/01 2009;94(1):103-110.

13. Filippidis AS, Phillips KR, Lopez-Rivera V, et al. Surgery in octogenarians with intracranial meningiomas improves functional outcome at 1 year. *Acta Neurochirurgica.* 2023 Oct 2023.

14. Flechl B, Ackerl M, Sax C, et al. Neurocognitive and sociodemographic functioning of glioblastoma long-term survivors. *Journal of Neuro-Oncology.* Sep 2012;109(2):331-339.

15. Greenberg E, Treger I, Ring H. Rehabilitation outcomes in patients with brain tumors and acute stroke - Comparative study of inpatient rehabilitation. *American Journal of Physical Medicine & Rehabilitation.* Jul 2006;85(7):568-573.

16. Hansen A, Pedersen CB, Minet LR, Beier D, Jarden JO, Søgaard K. Hemispheric tumor location and the impact on health-related quality of life, symptomatology, and functional performance outcomes in patients with glioma: an exploratory cross-sectional study. *Disabil Rehabil.* May 2021;43(10):1443-1449.

17. Heiland DH, Haaker G, Watzlawick R, et al. One decade of glioblastoma multiforme surgery in 342 elderly patients: what have we learned? *Journal of Neuro-Oncology.* Nov 2018;140(2):385-391.

18. Huang ME, Cifu DX, Keyser-Marcus L. Functional outcome after brain tumor and acute stroke: a comparative analysis. *Arch Phys Med Rehabil.* Nov 1998;79(11):1386-1390.

19. Huang ME, Wartella JE, Kreutzer JS. Functional outcomes and quality of life in patients with brain tumors: A preliminary report. *Archives of Physical Medicine and Rehabilitation.* Nov 2001;82(11):1540-1546.

20. Januel E, Ursu R, Alkhafaji A, et al. Impact of renin-angiotensin system blockade on clinical outcome in glioblastoma. *European Journal of Neurology.* Sep 2015;22(9):1304-1309.

21. Jones LW, Cohen RR, Mabe SK, et al. Assessment of physical functioning in recurrent glioma: preliminary comparison of performance status to functional capacity testing. *Journal of Neuro-Oncology.* Aug 2009;94(1):79-85.

22. Jones LW, Friedman AH, West MJ, et al. Quantitative assessment of cardiorespiratory fitness, skeletal muscle function, and body composition in adults with primary malignant glioma. *Cancer.* Feb 1 2010;116(3):695-704.

23. Jones LW, Mourtzakis M, Peters KB, et al. Changes in Functional Performance Measures in Adults Undergoing Chemoradiation for Primary Malignant Glioma: A Feasibility Study. *Oncologist.* 2010;15(6):636-647.

24. Joubert C, Sellier A, Sahuc P, et al. Neurosurgery for intracranial meningioma in patients aged more than 80 years: benefits and rationale. *British Journal of Neurosurgery.* Jul 2021;35(4):470-475.

25. Khan F, Amatya B. Use of the International Classification of Functioning, Disability and Health (ICF) to describe patient-reported disability in primary brain tumour in an Australian community cohort. *J Rehabil Med.* May 2013;45(5):434-445.

26. Khan F, Amatya B, Drummond K, Galea M. EFFECTIVENESS OF INTEGRATED MULTIDISCIPLINARY REHABILITATION IN PRIMARY BRAIN CANCER SURVIVORS IN AN AUSTRALIAN COMMUNITY COHORT: A CONTROLLED CLINICAL TRIAL. *Journal of Rehabilitation Medicine.* Sep 2014;46(8):754-760.

27. Kim BR, Chun MH, Han EY, Kim DK. Fatigue assessment and rehabilitation outcomes in patients with brain tumors. *Support Care Cancer.* Apr 2012;20(4):805-812.

28. Kim SH, Byun Y. Trajectories of Symptom Clusters, Performance Status, and Quality of Life During Concurrent Chemoradiotherapy in Patients With High-Grade Brain Cancers. *Cancer Nurs.* Jan/Feb 2018;41(1):E38-e47.

29. Kombos T, Picht T, Derdilopoulos A, Suess O. Impact of intraoperative neurophysiological monitoring on surgery of high-grade gliomas. *J Clin Neurophysiol.* Dec 2009;26(6):422-425.

30. Konglund A, Rogne SG, Lund-Johansen M, Scheie D, Helseth E, Meling TR. Outcome following surgery for intracranial meningiomas in the aging. *Acta Neurologica Scandinavica.* Mar 2013;127(3):161-169.

31. Kosteniuk SE, Gui C, Gariscsak PJ, Lau JC, Megyesi JF. Impact of Functional Magnetic Resonance Imaging on Clinical Outcomes in a Propensity-Matched Low Grade Glioma Cohort. *World Neurosurgery.* Dec 2018;120:E1143-E1148.

32. Krajewski S, Furtak J, Zawadka-Kunikowska M, Kachelski M, Birski M, Harat M. Comparison of the Functional State and Motor Skills of Patients after Cerebral Hemisphere, Ventricular System, and Cerebellopontine Angle Tumor Surgery. *International Journal of Environmental Research and Public Health.* Feb 2022;19(4).

33. Li HY, Huang HW, Zhang XK, et al. Postoperative Long-Term Independence Among the Elderly With Meningiomas: Function Evolution, Determinant Identification, and Prediction Model Development. *Frontiers in Oncology.* Mar 2021;11.

34. Mainio A, Hakko H, Niemelä A, Koivukangas J, Räsänen P. Depression and functional outcome in patients with brain tumors: a population-based 1-year follow-up study. *J Neurosurg.* Nov 2005;103(5):841-847.

35. Mainio A, Hakko H, Niemela A, Koivukangas J, Rasanen P. Gender difference in relation to depression and quality of life among patients with a primary brain tumor. *European Psychiatry.* Apr 2006;21(3):194-199.

36. Maschio M, Zarabla A, Maialetti A, et al. Quality of life, mood and seizure control in patients with brain tumor related epilepsy treated with lacosamide as add-on therapy: A prospective explorative study with a historical control group. *Epilepsy & Behavior.* Aug 2017;73:83-89.

37. Nakajima R, Kinoshita M, Okita H, Yahata T, Nakada M. Awake surgery for glioblastoma can preserve independence level, but is dependent on age and the preoperative condition. *Journal of Neuro-Oncology.* Aug 2019;144(1):155-163.

38. Nakajima R, Kinoshita M, Okita H, Nakada M. Glioblastomas at the white matter of temporo-parietal junction cause a poor postoperative independence level. *Journal of Neuro-Oncology.* 2023 Oct 2023.

39. Ness KK, Morris EB, Nolan VG, et al. Physical performance limitations among adult survivors of childhood brain tumors. *Cancer.* Jun 15 2010;116(12):3034-3044.

40. El Rahal A, Cipriani D, Fung C, et al. Hydrocephalus Shunting in Supratentorial Glioblastoma: Functional Outcomes and Management. *Frontiers in Oncology.* Feb 2022;12.

41. Roberts PS, Nuño M, Sherman D, et al. The Impact of Inpatient Rehabilitation on Function and Survival of Newly Diagnosed Patients With Glioblastoma. *Pm&R.* Jun 2014;6(6):514-521.

42. Sacko A, Hou MM, Temgoua M, et al. Evolution of the Karnosky Performance Status throughout life in glioblastoma patients. *J Neurooncol.* May 2015;122(3):567-573.

43. Sass D, Vera E, Choi A, et al. Evaluation of the key geriatric assessment constructs in primary brain tumor population - a descriptive study. *J Geriatr Oncol.* Nov 2022;13(8):1194-1202.

44. Schiavolin S, Mariniello A, Broggi M, DiMeco F, Ferroli P, Leonardi M. Preoperative nonmedical predictors of functional impairment after brain tumor surgery. *Supportive Care in Cancer.* Apr 2022;30(4):3441-3450.

45. Spychka RA, Purdy GM, Easaw JC, et al. Feasibility of performance-based functional assessment in brain tumour survivors. *European Journal of Cancer Care.* Jul 2020;29(4).

46. Tabouret E, Boucard C, Devillier R, et al. Neuro-oncological patients admitted in intensive-care unit: predictive factors and functional outcome. *Journal of Neuro-Oncology.* Mar 2016;127(1):111-117.

47. Tankumpuan T, Utriyaprasit K, Chayaput P, Itthimathin P. Predictors of physical functioning in postoperative brain tumor patients. *J Neurosci Nurs.* Feb 2015;47(1):E11-21.

48. Varedi M, Lu L, Phillips NS, et al. Balance impairment in survivors of pediatric brain cancers: risk factors and associated physical limitations. *J Cancer Surviv.* Apr 2021;15(2):311-324.

49. Zhang J, Chen YS, Li YP, et al. Postoperative functional status in patients with supratentorial superficial low-grade glioma. *World Journal of Surgical Oncology.* Oct 2017;15.

50. Mahoney FI, Barthel DW. Functional evaluation: the Barthel Index: a simple index of independence useful in scoring improvement in the rehabilitation of the chronically ill. *Maryland state medical journal.* 1965.

51. Shah S, Vanclay F, Cooper B. Improving the sensitivity of the Barthel Index for stroke rehabilitation. *Journal of Clinical Epidemiology.* 1989/01/01/ 1989;42(8):703-709.

52. Wang Y-C, Chang P-F, Chen Y-M, et al. Comparison of responsiveness of the Barthel Index and modified Barthel Index in patients with stroke. *Disability and Rehabilitation.* 2023/03/13 2023;45(6):1097-1102.

53. Brunnstrom S. Motor Testing Procedures in Hemiplegia: Based on Sequential Recovery Stages. *Physical Therapy.* 1966;46(4):357-375.

54. Shah SK, Harasymiw SJ, Stahl PL. Stroke Rehabilitation: Outcome Based on Brunnstrom Recovery Stages. *The Occupational Therapy Journal of Research.* 1986/11/01 1986;6(6):365-376.

55. Rappaport M, Hall KM, Hopkins K, Belleza T, Cope DN. Disability rating scale for severe head trauma: coma to community. *Arch Phys Med Rehabil.* Mar 1982;63(3):118-123.

56. Yamal JM, Aisiku IP, Hannay HJ, Brito FA, Robertson CS. Disability Rating Scale in the First Few Weeks After a Severe Traumatic Brain Injury as a Predictor of 6-Month Functional Outcome. *Neurosurgery.* Feb 16 2021;88(3):619-626.

57. Oken MM, Creech RH, Tormey DC, et al. Toxicity and response criteria of the Eastern Cooperative Oncology Group. *Am J Clin Oncol.* Dec 1982;5(6):649-655.

58. Azam F, Latif MF, Farooq A, et al. Performance Status Assessment by Using ECOG (Eastern Cooperative Oncology Group) Score for Cancer Patients by Oncology Healthcare Professionals. *Case Rep Oncol.* Sep-Dec 2019;12(3):728-736.

59. Young J, Badgery-Parker T, Dobbins T, et al. Comparison of ECOG/WHO Performance Status and ASA Score as a Measure of Functional Status. *Journal of Pain and Symptom Management.* 2015/02/01/ 2015;49(2):258-264.

60. Granger CV, Hamilton BB, Linacre JM, Heinemann AW, Wright BD. Performance Profiles of the Functional Independence Measure. *American Journal of Physical Medicine & Rehabilitation.* 1993;72(2):84-89.

61. Holden MK, Gill KM, Magliozzi MR, Nathan J, Piehl-Baker L. Clinical gait assessment in the neurologically impaired: reliability and meaningfulness. *Physical therapy.* 1984;64(1):35-40.

62. Rosanna Chau MW, Chan SP, Wong YW, Lau MYP. Reliability and validity of the Modified Functional Ambulation Classification in patients with hip fracture. *Hong Kong Physiotherapy Journal.* 2013/06/01/ 2013;31(1):41-44.

63. Keith RA, Granger CV, Hamilton BB, Sherwin FS. The functional independence measure: a new tool for rehabilitation. *Adv Clin Rehabil.* 1987;1:6-18.

64. Gosman-Hedström G, Svensson E. Parallel reliability of the functional independence measure and the Barthel ADL index. *Disabil Rehabil.* Nov 10 2000;22(16):702-715.

65. Nichol AD, Higgins AM, Gabbe BJ, Murray LJ, Cooper DJ, Cameron PA. Measuring functional and quality of life outcomes following major head injury: Common scales and checklists. *Injury.* 2011/03/01/ 2011;42(3):281-287.

66. Ring H, Feder M, Schwartz J, Samuels G. Functional measures of first-stroke rehabilitation inpatients: usefulness of the Functional Independence Measure total score with a clinical rationale. *Arch Phys Med Rehabil.* Jun 1997;78(6):630-635.

67. Hauser SL, Dawson DM, Lehrich JR, et al. Intensive Immunosuppression in Progressive Multiple Sclerosis. *New England Journal of Medicine.* 1983;308(4):173-180.

68. WHO. How to use the ICF: Apractical manual for using the International Classification of Functioning, Disability and Health (ICF). *Exposure draft for Comment*: World Health Organization; 2013.

69. Karnofsky D, Burchenal J. Evaluation of chemotherpeutic agents. *New York, NY, Columbia University.* 1949;19.

70. Péus D, Newcomb N, Hofer S. Appraisal of the Karnofsky Performance Status and proposal of a simple algorithmic system for its evaluation. *BMC Med Inform Decis Mak.* Jul 19 2013;13:72.

71. Schag CC, Heinrich RL, Ganz PA. Karnofsky performance status revisited: reliability, validity, and guidelines. *J Clin Oncol.* Mar 1984;2(3):187-193.

72. Rankin J. Cerebral vascular accidents in patients over the age of 60. II. Prognosis. *Scott Med J.* May 1957;2(5):200-215.

73. Pożarowszczyk N, Kurkowska-Jastrzębska I, Sarzyńska-Długosz I, Nowak M, Karliński M. Reliability of the modified Rankin Scale in clinical practice of stroke units and rehabilitation wards. *Front Neurol.* 2023;14:1064642.

74. Banks JL, Marotta CA. Outcomes validity and reliability of the modified Rankin scale: implications for stroke clinical trials: a literature review and synthesis. *Stroke.* Mar 2007;38(3):1091-1096.

75. Demeurisse G, Demol O, Robaye E. Motor evaluation in vascular hemiplegia. *Eur Neurol.* 1980;19(6):382-389.

76. Fayazi M, Dehkordi SN, Dadgoo M, Salehi M. Test-retest reliability of Motricity Index strength assessments for lower extremity in post stroke hemiparesis. *Med J Islam Repub Iran.* Feb 2012;26(1):27-30.

77. Sunderland A, Tinson D, Bradley L, Hewer RL. Arm function after stroke. An evaluation of grip strength as a measure of recovery and a prognostic indicator. *J Neurol Neurosurg Psychiatry.* Nov 1989;52(11):1267-1272.

78. Williams M. Manual muscle testing, development and current use. *Phys Ther Rev (1948).* Dec 1956;36(12):797-805.

79. Naqvi U, Sherman AL. Muscle strength grading. *StatPearls [Internet]*: StatPearls Publishing; 2023.

80. Bleehen N, Stenning S. A Medical Research Council trial of two radiotherapy doses in the treatment of grades 3 and 4 astrocytoma. *British journal of cancer.* 1991;64(4):769-774.

81. Hlatky MA, Boineau RE, Higginbotham MB, et al. A brief self-administered questionnaire to determine functional capacity (the Duke Activity Status Index). *Am J Cardiol.* Sep 15 1989;64(10):651-654.

82. Li MHG, Bolshinsky V, Ismail H, Ho K-M, Heriot A, Riedel B. Comparison of Duke Activity Status Index with cardiopulmonary exercise testing in cancer patients. *Journal of Anesthesia.* 2018/08/01 2018;32(4):576-584.

83. Jette AM, Davies AR, Cleary PD, et al. The Functional Status Questionnaire: reliability and validity when used in primary care. *J Gen Intern Med.* May-Jun 1986;1(3):143-149.

84. Rosow I, Breslau N. A Guttman health scale for the aged. *J Gerontol.* Oct 1966;21(4):556-559.

85. Bergner M, Bobbitt RA, Carter WB, Gilson BS. The Sickness Impact Profile: development and final revision of a health status measure. *Med Care.* Aug 1981;19(8):787-805.

86. Schepers VPM, Ketelaar M, Visser-Meily JMA, Dekker J, Lindeman E. Responsiveness of functional health status measures frequently used in stroke research. *Disability and Rehabilitation.* 2006/01/01 2006;28(17):1035-1040.

87. Üstün TB. *Measuring health and disability: Manual for WHO disability assessment schedule WHODAS 2.0.* World Health Organization; 2010.

88. Federici S, Bracalenti M, Meloni F, Luciano JV. World Health Organization disability assessment schedule 2.0: An international systematic review. *Disability and Rehabilitation.* 2017/11/06 2017;39(23):2347-2380.

89. Brzycki M. Strength Testing—Predicting a One-Rep Max from Reps-to-Fatigue. *Journal of Physical Education, Recreation & Dance.* 1993/01/01 1993;64(1):88-90.

90. Cheng DK, Nelson M, Brooks D, Salbach NM. Validation of stroke-specific protocols for the 10-meter walk test and 6-minute walk test conducted using 15-meter and 30-meter walkways. *Top Stroke Rehabil.* May 2020;27(4):251-261.

91. Butland RJ, Pang J, Gross ER, Woodcock AA, Geddes DM. Two-, six-, and 12-minute walking tests in respiratory disease. *Br Med J (Clin Res Ed).* May 29 1982;284(6329):1607-1608.

92. Mathiowetz V, Volland G, Kashman N, Weber K. Adult norms for the Box and Block Test of manual dexterity. *Am J Occup Ther.* Jun 1985;39(6):386-391.

93. Bovend'Eerdt TJ, Dawes H, Johansen-Berg H, Wade DT. Evaluation of the Modified Jebsen Test of Hand Function and the University of Maryland Arm Questionnaire for Stroke. *Clin Rehabil.* Mar 2004;18(2):195-202.

94. Johansson GM, Häger CK. A modified standardized nine hole peg test for valid and reliable kinematic assessment of dexterity post-stroke. *Journal of NeuroEngineering and Rehabilitation.* 2019/01/14 2019;16(1):8.

95. Watson MJ. Refining the Ten-metre Walking Test for Use with Neurologically Impaired People. *Physiotherapy.* 2002;88(7):386-397.

96. Peters DM, Fritz SL, Krotish DE. Assessing the reliability and validity of a shorter walk test compared with the 10-Meter Walk Test for measurements of gait speed in healthy, older adults. *J Geriatr Phys Ther.* Jan-Mar 2013;36(1):24-30.

97. Scivoletto G, Tamburella F, Laurenza L, Foti C, Ditunno JF, Molinari M. Validity and reliability of the 10-m walk test and the 6-min walk test in spinal cord injury patients. *Spinal Cord.* 2011/06/01 2011;49(6):736-740.

98. Jones CJ, Rikli RE, Beam WC. A 30-s chair-stand test as a measure of lower body strength in community-residing older adults. *Res Q Exerc Sport.* Jun 1999;70(2):113-119.

99. Fisher AG, Liu Y, Velozo CA, Pan AW. Cross-cultural assessment of process skills. *Am J Occup Ther.* Oct 1992;46(10):876-885.

100. Bray K, Fisher AG, Duran L. The validity of adding new tasks to the assessment of motor and process skills. *Am J Occup Ther.* Jul-Aug 2001;55(4):409-415.

101. Astrand PO, Ryhming I. A nomogram for calculation of aerobic capacity (physical fitness) from pulse rate during sub-maximal work. *J Appl Physiol.* Sep 1954;7(2):218-221.

102. Berg K, Wood-Dauphine S, Williams J, Gayton D. Measuring balance in the elderly: preliminary development of an instrument. *Physiotherapy Canada.* 1989;41(6):304-311.

103. Azuma Y, Chin T, Miura Y. The relationship between balance ability and walking ability using the Berg Balance Scale in people with transfemoral amputation. *Prosthet Orthot Int.* Aug 2019;43(4):396-401.

104. Berg KO, Wood-Dauphinee SL, Williams JI, Maki B. Measuring balance in the elderly: validation of an instrument. *Can J Public Health.* Jul-Aug 1992;83 Suppl 2:S7-11.

105. Berg K, Wood-Dauphinee S, Williams J. The Balance Scale: reliability assessment with elderly residents and patients with an acute stroke. *Scandinavian journal of rehabilitation medicine.* 1995;27(1):27-36.

106. Wee JY, Wong H, Palepu A. Validation of the Berg Balance Scale as a predictor of length of stay and discharge destination in stroke rehabilitation. *Arch Phys Med Rehabil.* May 2003;84(5):731-735.

107. Jones LW, Eves ND, Mackey JR, et al. Safety and feasibility of cardiopulmonary exercise testing in patients with advanced cancer. *Lung Cancer.* 2007/02/01/ 2007;55(2):225-232.

108. Massy-Westropp NM, Gill TK, Taylor AW, Bohannon RW, Hill CL. Hand Grip Strength: age and gender stratified normative data in a population-based study. *BMC Res Notes.* Apr 14 2011;4:127.

109. Vaishya R, Misra A, Vaish A, Ursino N, D'Ambrosi R. Hand grip strength as a proposed new vital sign of health: a narrative review of evidences. *J Health Popul Nutr.* Jan 9 2024;43(1):7.

110. Labott BK, Bucht H, Morat M, Morat T, Donath L. Effects of Exercise Training on Handgrip Strength in Older Adults: A Meta-Analytical Review. *Gerontology.* 2019;65(6):686-698.

111. Reuben DB, Siu AL. An objective measure of physical function of elderly outpatients. The Physical Performance Test. *J Am Geriatr Soc.* Oct 1990;38(10):1105-1112.

112. Guralnik JM, Simonsick EM, Ferrucci L, et al. A short physical performance battery assessing lower extremity function: association with self-reported disability and prediction of mortality and nursing home admission. *J Gerontol.* Mar 1994;49(2):M85-94.

113. Pavasini R, Guralnik J, Brown JC, et al. Short Physical Performance Battery and all-cause mortality: systematic review and meta-analysis. *BMC Medicine.* 2016/12/22 2016;14(1):215.

114. Sandin KJ, Smith BS. The measure of balance in sitting in stroke rehabilitation prognosis. *Stroke.* Jan 1990;21(1):82-86.

115. Bohannon R. Correlation of lower limbs strengths and other variables with standing Performance in stroke patients. *Physiotherapy Canada.* 1989;41:198-201.

116. Podsiadlo D, Richardson S. The timed "Up & Go": a test of basic functional mobility for frail elderly persons. *J Am Geriatr Soc.* Feb 1991;39(2):142-148.

117. Andersen LB. A maximal cycle exercise protocol to predict maximal oxygen uptake. *Scand J Med Sci Sports.* Jun 1995;5(3):143-146.
